# Biological and Molecular Effects of *Trypanosoma cruzi* Residence in a LAMP-Deficient Intracellular Environment

**DOI:** 10.3389/fcimb.2021.788482

**Published:** 2022-01-06

**Authors:** Anny Carolline Silva Oliveira, Luisa Rezende, Vladimir Gorshkov, Marcella Nunes Melo-Braga, Thiago Verano-Braga, Weslley Fernandes-Braga, Jorge Luís de Melo Guadalupe, Gustavo Batista de Menezes, Frank Kjeldsen, Hélida Monteiro de Andrade, Luciana de Oliveira Andrade

**Affiliations:** ^1^ Department of Morphology, Biological Sciences Institute—ICB, Federal University of Minas Gerais (UFMG), Belo Horizonte, Brazil; ^2^ Protein Research Group, Department of Biochemistry and Molecular Biology, University of Southern Denmark, Odense, Denmark; ^3^ Department of Biochemistry and Immunology, Biological Sciences Institute—ICB, Federal University of Minas Gerais (UFMG), Belo Horizonte, Minas Gerais, Brazil; ^4^ Hypertension Lab/Functional Proteomics Group, Department of Physiology and Biophysics, Biological Sciences Institute—ICB, Federal University of Minas Gerais (UFMG), Belo Horizonte, Brazil; ^5^ Laboratory of Leishmanioses, Department of Parasitology, Biological Sciences Institute—ICB, Federal University of Minas Gerais (UFMG), Belo Horizonte, Brazil

**Keywords:** *Trypanosoma cruzi*, Chagas disease, LAMP proteins, parasite invasion, intracellular environment, proteomic analysis, TMT (tandem mass tags)

## Abstract

*Trypanosoma cruzi* invades non-professional phagocytic cells by subverting their membrane repair process, which is dependent on membrane injury and cell signaling, intracellular calcium increase, and lysosome recruitment. Cells lacking lysosome-associated membrane proteins 1 and 2 (LAMP1 and LAMP2) are less permissive to parasite invasion but more prone to parasite intracellular multiplication. Several passages through a different intracellular environment can significantly change *T. cruzi*’s gene expression profile. Here, we evaluated whether one single passage through LAMP-deficient (KO) or wild-type (WT) fibroblasts, thus different intracellular environments, could influence *T. cruzi* Y strain trypomastigotes’ ability to invade L6 myoblasts and WT fibroblasts host cells. Parasites released from LAMP2 KO cells (TcY-L2^−/−^) showed higher invasion, calcium signaling, and membrane injury rates, for the assays in L6 myoblasts, when compared to those released from WT (TcY-WT) or LAMP1/2 KO cells (TcY-L1/2^−/−^). On the other hand, TcY-L1/2^−/−^ showed higher invasion, calcium signaling, and cell membrane injury rates, for the assays in WT fibroblasts, compared to TcY-WT and TcY-L1/2^−/−^. Albeit TcY-WT presented an intermediary invasion and calcium signaling rates, compared to the others, in WT fibroblasts, they induced lower levels of injury, which reinforces that signals mediated by surface membrane protein interactions also have a significant contribution to trigger host cell calcium signals. These results clearly show that parasites released from WT or LAMP KO cells are distinct from each other. Additionally, these parasites’ ability to invade the cell may be distinct depending on which cell type they interact with. Since these alterations most likely would reflect differences among parasite surface molecules, we also evaluated their proteome. We identified few protein complexes, membrane, and secreted proteins regulated in our dataset. Among those are some members of MASP, mucins, trans-sialidases, and gp63 proteins family, which are known to play an important role during parasite infection and could correlate to TcY-WT, TcY-L1/2^−/−^, and TcY-L2^−/−^ biological behavior.

## Introduction

*Trypanosoma cruzi* is an important human pathogen that causes the American trypanosomiasis or Chagas disease—a tropical neglected disease estimated to affect about 6–7 million people worldwide (WHO, 2021). *T. cruzi*’s infective trypomastigote forms invade non-professional phagocytic cells through a process dependent on parasite interaction with host cells and activation of calcium signaling pathways that lead to parasite internalization (Andrade and Andrews, 2005; de Souza et al., 2010; Fernandes and Andrews, 2012). For this invasion process, *T. cruzi* subverts plasma membrane repair mechanism through promoting small wounds at the host cell plasma membrane and by engaging host cell surface receptors. Both events trigger signaling pathways, which lead to intracellular calcium increase in the host cell (Burleigh and Woolsey, 2002) and promote lysosomal recruitment and fusion at the parasite contact site (Tardieux et al., 1992), triggering a compensatory endocytosis process that leads to parasite internalization into the host cell (Tam et al., 2010; Fernandes et al., 2011; Fernandes and Andrews, 2012). Other lysosomes fuse to this newly formed parasitophorous vacuole (Fernandes et al., 2011) guaranteeing parasite’s retention in the host cell cytoplasm (Andrade and Andrews, 2004).

In the last decade, our group has shown that cells deficient in two lysosomal membrane glycoproteins, both rich in sialic acid and known as lysosome-associated membrane protein (LAMP), LAMP-1 and LAMP-2 (Kornfeld and Mellman, 1989), are less permissive to *T. cruzi* invasion but more prone to parasite intracellular development (Albertti et al., 2010). We have also shown that LAMP-2 absence alone is sufficient for the decreased parasite invasion rate observed in LAMP-1/2-deficient cells (LAMP1/2^−/−^) (Couto et al., 2017). The latter is due to the compromised ability of cells deficient in LAMP-2 in performing plasma membrane repair (Couto et al., 2017). Even though lysosomal exocytosis is normal in these cells, the compensatory endocytosis triggered by these exocytic events is compromised and therefore also parasite internalization (Couto et al., 2017). LAMP-2 proteins were shown to participate in the endosomal/lysosomal cholesterol export (Schneede et al., 2011). Therefore, LAMP-2 deficiency leads to accumulation of cholesterol in endo-/lysosomal compartments and, consequently, lower levels of cholesterol content in their plasma membrane (Schneede et al., 2011; Couto et al., 2017). This, in turn, leads to less caveolin at the cell surface, interfering with the compensatory endocytosis process, imperative for the membrane repair process and parasite internalization (Couto et al., 2017). Aside from cholesterol accumulation in endo-/lysosomal compartments, cells lacking LAMP-2 or both LAMPs also accumulated autophagic vacuoles in their cytoplasm at different degrees (Eskelinen et al., 2004), therefore creating a different intracellular environment.

Changes in the intracellular environment have been previously shown to interfere with parasite gene expression profile. Dos Santos et al. (2012) showed that trypomastigotes derived from distinct cell types presented a differential expression profile of the MASP surface protein family (dos Santos et al., 2012). For this study, they used *T. cruzi* CL Brener trypomastigote forms obtained after 17 weekly maintenance passages in L6 myoblasts or LLC-MK2 epithelial cells. This differential profile of MASP expression is also reflected in differences in parasite invasion ability (dos Santos et al., 2012). As mentioned before, cells lacking LAMP-2 protein present higher cholesterol content in their cytoplasm. In addition, in these cells, *T. cruzi* intracellular multiplication rates are higher when compared to the rates observed for control cells. Thus, in the present work, we evaluated whether one passage through wild-type (WT) and LAMP-deficient fibroblasts, thus different intracellular environments, could also induce differences in parasite biological and biochemical parameters, such as protein expression profile, consequently influencing parasite invasion. For this, *T. cruzi* Y strain trypomastigotes obtained from LLC-MK2 epithelial cells were used to infect WT, LAMP1/2^−/−^, and LAMP2^−/−^ cells, from which cultures recovered trypomastigotes were evaluated concerning their ability to adhere, invade, trigger intracellular calcium signaling, and induce membrane injury in L6 myoblasts and wild-type fibroblasts. In addition, through a proteomic approach, we also evaluated the newly released trypomastigotes’ membrane and regulated subproteomes and their correlations to the parasites’ biological characteristics and behavior.

## Material and Methods

### *Trypanosoma cruzi* and Cell Cultures

Mouse fibroblasts cell lines, derived from WT, LAMP-1/2^−/−^, or LAMP-2^−/−^ knockout C57BL/6J mice, were obtained from a collection of cell lines from Dr. Paul Saftig’s laboratory (Biochemisches Institut/Christian-Albrechts-Universität Kiel, Germany), which were previously generated by spontaneous immortalization of primary fibroblasts in culture around passages 10–20 (Eskelinen et al., 2004). The cells were maintained in high-glucose Dulbecco’s modified Eagle’s medium (DMEM) (Thermo Fischer Scientific) supplemented with 10% fetal bovine serum (FBS), 1% penicillin/streptomycin (100 U/ml and 100 μg/ml, respectively) and 1% glutamine (DMEM 10%).

Tissue culture trypomastigotes from the *T. cruzi* Y strain were obtained and purified from the supernatant of infected LLC-MK2 monolayers, as described previously (Andrews et al., 1987), and used to infect wild-type or LAMP-deficient fibroblasts.

### *Trypanosoma cruzi* Invasion Assays

For invasion assays, 5 × 10^4^ cells (WT fibroblasts or L6 murine myoblasts) in high-glucose DMEM 10% were plated per well on a 24-well plate, containing 13-mm round glass coverslips. Cells were plated 24 h before the experiment and incubated at 37°C and 5% CO_2_. Cells were then exposed to *T. cruzi* Y strain trypomastigotes obtained from wild-type (TcY-WT), LAMP-2-deficient (TcY-L2^−/−^), or LAMP-1- and LAMP-2-deficient (TcY-L1/2^−/−^) fibroblasts for 40 min at 37°C at a multiplicity of infection (MOI) of 50. After parasite exposure, the monolayers were washed four times with phosphate-buffered saline containing Ca^2+^ and Mg^2+^ (PBS^+/+^), in order to remove the non-internalized parasites, and fixed in 4% paraformaldehyde overnight. After fixation, cells were processed for immunofluorescence.

### Immunofluorescence and Quantification of Parasite Invasion

After fixation, coverslips with attached cells were washed three times in PBS, incubated for 20 min with PBS containing 2% bovine serum albumin (BSA) and processed for an inside/outside immunofluorescence invasion assay as described previously (Andrews et al., 1987). Briefly, extracellular parasites were immunostained with rabbit anti-*T. cruzi* polyclonal antibodies in a 1:500 dilution in PBS/BSA for 1 h at room temperature (RT) and washed and labeled with Alexa Fluor-546 conjugated anti-rabbit IgG antibody (Thermo Fischer Scientific) in a proportion of 1:500 in PBS/BSA for 45 min. After that, the DNA of host cells and parasites was stained for 1 min with DAPI (4′,6-diamidino-2-phenylindole, dihydrochloride, Sigma), 0.1 μM in PBS, mounted, and examined on a ZEISS Axio Vert.A1 microscope.

### *Trypanosoma cruzi* Adhesion Assays

The assays followed the protocol previously described by Schenkman, Diaz, and Nussenzweig (1991) (Schenkman et al., 1991). Briefly, WT fibroblasts or L6 myoblasts were plated in each well of a 24-well plate, containing 13-mm round glass coverslips, 24 h before the experiment and incubated at 37°C and 5% CO_2_. Cells were then washed with Hank’s balanced salt solution (HBSS, Sigma), pre-fixed with 2% glutaraldehyde in PBS^+/+^ for 5 min at 4°C and incubated for 24 h in 0.16M pH 8.3 ethanolamine solution. Before parasite exposure, cells were washed two times with DMEM containing 0.2% BSA. Cells were exposed to TcY-WT, TcY-L2^−/−^, and TcY-L1/2^−/−^ trypomastigotes at 37°C and 5% CO_2_ for 40 min at an MOI of 50. After exposure, cells were washed with PBS, fixed with 4% paraformaldehyde (PFA), and stained using rapid panotic staining. Attached parasites were counted using a microscope.

### Host Cell Membrane Injury Assays

WT fibroblasts or L6 myoblasts were cultured in a 24-well plate (5 × 10^4^ cells/well) at 37°C in 5% CO_2_ in DMEM 10%. After 24 h, cell monolayers were exposed to DMEM 10% containing PI (10 μg/ml) in the presence or absence of TcY-WT, TcY-L2^−/−^, or TcY-L1/2^−/−^ trypomastigotes for 30 min at 37°C at an MOI of 100. Alternatively, cells were exposed to parasites for 40 min, washed three times with PBS^+/+^, and then exposed to PI staining for another 40 min. All cells were washed twice with PBS^+/+^, trypsinized, and analyzed by flow cytometry (FACS Scan; Becton Dickinson). The data were analyzed using FlowJo v10.1 software (Tree Star, Inc.).

### Calcium Signaling Assays

For the calcium signaling assays, 2.5 × 10^4^ cells (WT fibroblasts or L6 myoblasts) in high-glucose DMEM 10% were plated in each well of four-well confocal glass-bottom dishes and, 24 h later, incubated with a fluorescent calcium probe [5 μM Fluo-4/AM (Thermo Fisher), 0.01% pluronic acid and 2.0 mM probenecid (Sigma), for 50 min at 37°C (Luo et al., 2011). Afterwards, cells were exposed to the following conditions: (1) culture medium; (2) ionomicin (5 μM); and (3) TcY-WT, TcY-L2^−/−^, or TcY-L1/2^−/−^ trypomastigotes at an MOI of 100. Cells were analyzed in a Nikon C2 Plus confocal microscope using the time lapsing resource and photographed for 600 s without intervals. The changes observed for the intracellular calcium concentration were expressed as the probe intensity fluorescence fold change accordingly with the equation (F/F_0_), F being the maximum fluorescence intensity observed and F_0_ the basal fluorescence observed for the point 0 (Pinto et al., 2013; Oliveira et al., 2015).

### Gelatin Zymography Assays

To evaluate proteolytic activity in TcY-WT, TcY-L1/2^−/−^, and TcY-L2^−/−^ trypomastigotes, cellular extracts were prepared using 5 × 10^8^ trypomastigotes (70% pure regarding amastigote forms contamination) in lysis buffer [5 mM Tris–HCl pH 8.0, 10% glycerol, 1 mM EDTA (Sigma), 1 mM DTT (Promega), 10 mM cOmplete Mini EDTA-free protease inhibitors (Roche)] and protein concentration quantified by Bradford’s method (Bradford, 1976). Gelatin zymography assays were performed as previously described (Leber and Balkwill, 1997) with few adaptations. Briefly, 12.5 μg of total protein were mixed with loading buffer without reducing agents and electrophoresed through a 12% polyacrylamide gel copolymerized with gelatin (Sigma) at a final concentration of 1 mg/ml. Gels were soaked in renaturing buffer [50 mM Tris–HCl, pH 7.5; 2.5% Triton X-100 (Sigma)] for 30 min at (RT), followed by one wash for 30 min at (RT) in development buffer (50 mM Tris–HCl, 200 mM NaCl, 5 mM CaCl_2_, pH 7.5) and incubated for 24 h in development buffer at 37°C. Gels were stained with R-250 Coomassie brilliant blue (Sigma). Gelatinolytic activities were detected as pale bands against the dark blue background. Band intensities were quantified using ImageJ software and expressed as the pixels fold change regarding TcY-WT pixels read mean. The molecular weight standard was purchased from BioRad Prestained SDS-PAGE Standards, Broad Range.

### Enzymatic Kinetics Assays

To evaluate the cysteine and serine proteolytic activities in TcY-WT, TcY-L1/2^−/−^, and TcY-L2^−/−^ trypomastigotes, cellular extracts were prepared as described above and 5 μg of total protein extract mixed with 50 μM N-acetyl-Tyr-Val-Ala-Asp-7-amido-4-trifluoromethylcoumarin (Ac-YVAD-AFC #A9965, Sigma-Aldrich) caspase-like or 13 μM N-succinyl-Leu-Leu-Val-Tyr 7-Amido-4-Methylcoumarin (Suc-LLVY-AMC, #S6510—Sigma-Aldrich) chymotrypsin-like fluorogenic substrates. The reactions were performed in buffer containing 50 mM Tris–HCl, pH 8.0, 10 mM MgCl_2_, and 1 mM DTT, in the presence or absence of 20 μM MG-132 (#M7449, Sigma-Aldrich) inhibitor, and incubated at 37°C from 0 to 120 min with readings every 20 min. The readings were performed in the Synergy 2 Multi-detection Microplate (BioTek Instruments, Inc.) with excitation wavelength of 380/20 and emission wavelengths of 508/20 or 440/40 for Ac-YVAD-AFC or Suc-LLVY-AMC substrates, respectively.

### Membrane Proteins Fraction and Sample Preparation for MS/MS Analysis

To obtain the membrane proteins fraction, about 1 × 10^8^ trypomastigotes forms released from wild-type or LAMP-deficient fibroblasts, presenting about 70%–80% purity regarding amastigote forms contamination, were centrifuged during 10 min at 2,200*g* at 4°C and washed two times with PBS (pH 7.2). Samples were resuspended in 0.1M sodium carbonate lysis buffer (pH 11.5) supplemented with protease inhibitor cocktail (Protease Inhibitor Mix, GE Healthcare, USA), submitted to two ultrasonic pulses during 20 s on ice and incubated for 1 h on ice before ultracentrifugation at 150,000*g* for 90 min at 4°C (Sorvall Ultra Pro 80 Ultracentrifuge). Pellets were washed one time with 500 mM triethylammonium bicarbonate (TEAB) and one time with 50 mM TEAB.

Samples were suspended in 6M urea, 2M thiourea, and 50 mM TEAB buffer and reduced with 10 mM dithiothreitol (DTT) at (RT) for 1 h. After reduction, free thiols were alkylated with 20 mM iodoacetamide (IAA) in the dark for 1 h (RT). Samples were digested for 3 h (RT) in 1:100 ratio with Lys-C. Afterwards, samples were diluted eightfold with 50 mM TEAB (pH 8.0) and proteins digested with 1:100 ratio trypsin (Promega, Madison, WI) for overnight at 37°C. The reaction was quenched by adding formic acid (FA; 1% final concentration, Sigma-Aldrich, St. Louis, MO) followed by centrifugation at 14,000*g* for 10 min.

### Peptide TMT Labeling

The dried and digested peptides were resuspended in 100 mM HEPES (pH 8.5) with about 10 μg of peptide, as determined using a Qubit Protein Assay Kit (Thermo Fisher), from each sample used for TMT labeling. Six tags (tags 126, 127N, 128N, 129N, 130N, and 131N) from a TMT 11-plex kit (Thermo Fisher) were used to label the samples from each biological replicate. Aliquots (0.8 mg) of each reagent in 41 μl of anhydrous acetonitrile were incubated with peptide samples for 2 h at RT. The reaction was quenched by the addition of 8 μl of 5% hydroxylamine followed by incubation for 15 min at R. For each biological replicate, equal amounts of the six TMT-labeled samples were mixed, and the TMT-labeled peptides were purified on an Oasis HLB 10 mg cartridge (Waters Corporation), which had been wetted with 100% acetonitrile and equilibrated twice with 0.1% aqueous trifluoroacetic acid (TFA). Adsorbed peptides were washed with 2 ml 0.1% TFA twice, eluted in 300 μl of 30% acetonitrile and 300 μl of 60% acetonitrile, and dried using a SpeedVac evaporator. Afterwards, the dried TMT-labeled peptides were resuspended in 2% acetonitrile in 10 mM ammonium formate (pH 9.3) for high-pH reverse phase chromatography.

### High-pH Reverse Phase Chromatography

TMT-labeled peptides were injected onto an C18 Acquity UPLC MClass CSH 1.7 µm, 300 µm × 100 mm (Waters), using a Dionex Ultimate 3000 HPLC system. Buffer A was composed of 2% acetonitrile in 10 mM ammonium formate (pH 9.3) and buffer B of 80% acetonitrile in 10 mM ammonium formate (pH 9.3). Columns were run at 5 µl/min at 30°C, starting at 2% buffer B, and rising to 40% B over the course of a 27-min linear gradient. Buffer B was increased to 50% for 4 min; to 70% for 4 min; to 95% for 10 min, followed by a drop back to 2% B for 1 min, which was maintained until the end of the run at 71 min. Fractions were collected from the 4th to the 44th min with 180 s per fraction, producing 30 fractions. Fractions were concatenated into 10 samples, for example, the 1st, 11th, and 21st fractions were pooled in the same well of a 96-well plate. The 11 samples (10 samples plus the flow through—peptides eluted in the beginning of the starting conditions of high pH separation) were dried using a SpeedVac evaporator and solubilized in 0.1% FA.

### LC–MS/MS and Data Analysis

Samples were analyzed by an EASY-nano LC system (Proxeon Biosystems, Odense, Denmark) coupled online to an Q Exactive HF—Orbitrap mass spectrometer (QE-HF) (Thermo Scientific, Waltham, USA). Peptides from each fraction (2 µg each) were loaded onto a 18-cm fused silica column with integrated emitter (75 µm inner diameter) packed in-house with reverse phase resin ReproSil-Pur C18-AQ 3 µm (Dr. Maisch GmbH, Germany) and eluted using a gradient from 97% phase A (0.1% FA) to 28% phase B (0.1% FA, 95% acetonitrile) for 64 min, for all 11 fractions, 28–45% phase B for 10 min, 45–100% phase B for 3 min, and 100% phase B for 8 min in a total of 86 min at 250 nl/min. After each run, the column was washed with phase B and re-equilibrated with phase A. Mass spectra were acquired in positive ion mode applying data-dependent acquisition. Each MS scan in the Orbitrap (mass range of m/z of 350–1,500 and resolution 120,000 at *m/z* 200) was followed by MS/MS of the 20 most intense ions (Top 20). Fragmentation was performed by higher energy collisional dissociation (HCD), and selected ions were dynamically excluded for 20 s. Raw data were viewed in Xcalibur v.4.2 (Thermo Scientific, Waltham, USA). Data processing was performed using Proteome Discoverer v.2.4 (Thermo Scientific, Waltham, USA), by searching raw files with SequestHT algorithm against a *T. cruzi* Y strain database (release 47, downloaded in May 2020), containing the proteins of the parasite found in the newly sequenced *T. cruzi* Y strain (Callejas-Hernandez et al., 2018). Common contaminant proteins were also added to the database, and all contaminant proteins identified were filtered and removed from the result lists. The searches were performed with the following parameters: MS accuracy, 10 ppm; MS/MS accuracy, 0.02 Da; trypsin digestion with up to two missed cleavage allowed, carbamidomethyl modification of cysteine and peptide N-terminus TMT-6plex modification as fixed modifications and oxidized methionine and lysine modification by TMT-6plex as variable modifications. Number of proteins, protein groups, and number of peptides were filtered for false discovery rate (FDR) <1%, peptides with rank 1 and proteins with at least 2 peptides using Proteome Discoverer. ProteinCenter software (Thermo Scientific, Waltham, USA) was used to generate FASTA formatted files of groups of proteins of interest. Proteins were considered as regulated when their log_2_ fold change was higher than the mean plus two times the standard deviation or lower than the mean minus two times the standard deviation and their coefficient of variation lower than 40%. Transmembrane domain predictions were performed using TMHMM v.2.0 (https://services.healthtech.dtu.dk/service.php?TMHMM-2.0) (Sonnhammer et al., 1998; Krogh et al., 2001), and glycosylphosphatidylinositol (GPI) prediction was performed using the web tools GPI-SOM (http://gpi.unibe.ch/) and big-PI Predictor (https://mendel.imp.ac.at/gpi/gpi_server.html) (Eisenhaber et al., 1998; Sunyaev et al., 1999; Eisenhaber et al., 1999; Eisenhaber et al., 2000). DeepLoc (http://www.cbs.dtu.dk/services/DeepLoc/index.php) (Almagro Armenteros et al., 2017) was used to predict the proteins subcellular localization. SignalP v.5.0 (https://services.healthtech.dtu.dk/service.php?SignalP-5.0) (Almagro Armenteros et al., 2019) was used to predict proteins secreted by classical ER/Golgi pathway, and SecretomeP v.2.0 (http://www.cbs.dtu.dk/services/SecretomeP/) (Bendtsen et al., 2005) was used to predict non-classical protein secretion. Overrepresented categories were investigated through Gene Ontology (GO) enrichment analysis using TriTrypDB (http://tritrypdb.org/tritrypdb/), and redundant GO terms generated were summarized in clusters by REVIGO tool (http://revigo.irb.hr/) (Supek et al., 2011).

### Repository Data

The mass spectrometry proteomics data have been deposited to the ProteomeXchange Consortium (Deutsch et al., 2020) *via* the PRIDE (Perez-Riverol et al., 2019) partner repository with the dataset identifier PXD028897.

### Statistical Methods

Quantitative data were expressed as mean ± standard deviation (SD) of three independent experiments unless otherwise indicated. Statistical analysis was carried out using GraphPad Prism 7.0 software *via* one-way ANOVA followed by Fisher’s least significant difference (LSD) test or by controlling the false discovery rate (FDR) using the original procedure of Benjamini and Hochberg for multiple comparisons. Means were considered significantly different when p <0.05.

## Results

### The Residence in a LAMP-Deficient Intracellular Environment Modifies *T. cruzi* Invasion Profile

*T. cruzi* multiplication is increased in a LAMP-deficient environment (LAMP1/2^−/−^ or LAMP2^−/−^ fibroblasts) (Albertti et al., 2010), which suggests that differences in the intracellular environment can modify parasite biological behavior inside the host cell. In order to evaluate whether this LAMP-deficient environment may also influence the biological behavior of parasites released from these cells, *T. cruzi* Y strain trypomastigotes collected from a single infection cycle in WT (TcY-WT), LAMP1/2^−/−^ (TcY-L1/2^−/−^) or LAMP2^−/−^ (TcY-L2^−/−^) fibroblasts were evaluated concerning their ability to invade L6 myoblasts and WT fibroblasts. For this, these cells were exposed to TcY-WT, TcY-L1/2^−/−^, and TcY-L2^−/−^ infective trypomastigote forms at an MOI of 50 for 40 min.

**Figure 1:**
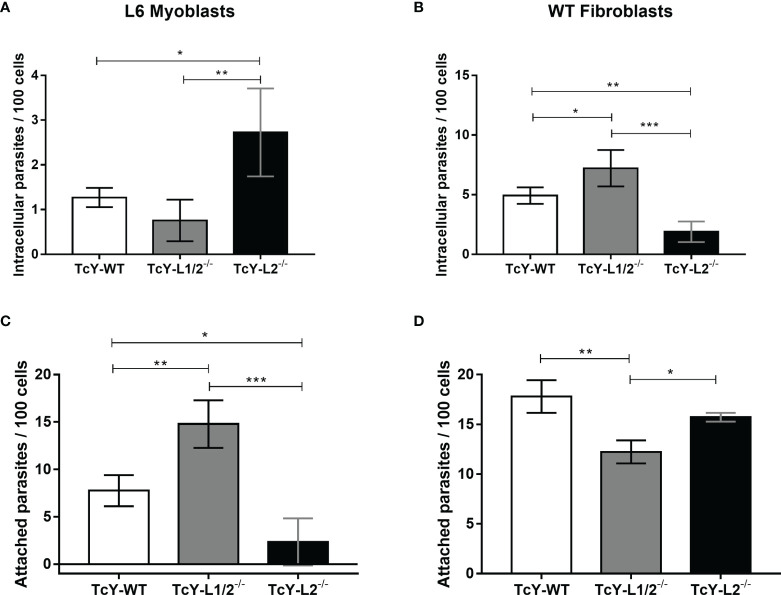
Parasite invasion and adhesion rates are altered by passage through WT or LAMP deficient fibroblasts but do not correlate to each other. Invasion assays **(A, B)**. L6 myoblasts **(A)** or WT fibroblasts **(B)** were exposed to TcY-WT, TcY-L1/2^−/−^, and TcY-L2^−/−^ trypomastigotes for 40 min at an MOI of 50. Cells were fixed with 4% PFA and labeled with *T. cruzi* antibody and DAPI. Adhesion assays **(C, D)**. L6 myoblasts **(C)** or WT fibroblasts **(D)** were fixed with 2% glutaraldehyde prior to incubation with to TcY-WT, TcY-L1/2^−/−^, and TcY-L2^−/−^ trypomastigotes for 40 min at MOI of 50. Cells were fixed with 4% PFA and stained with rapid panotic staining. Asterisks represent statistical significance between groups (*p < 0.05, **p < 0.01, ***p < 0.001, ****p < 0.0001, One-way ANOVA, Fisher’s LSD).

For L6 myoblasts, TcY-L2^−/−^ parasites presented the highest invasion rates, as evidenced by the number of intracellular parasites per 100 cells, when compared to TcY-WT and TcY-L1/2^−/−^ parasites, which did not show statistically significant differences between them (**Figure 1A**). On the other hand, for WT fibroblasts, TcY-L2^−/−^ parasites presented the lowest invasion rates, when compared to the other parasites, TcY-L1/2^−/−^ and TcY-WT (**Figure 1B**). Additionally, for these fibroblasts, TcY-WT invasion rate was higher than that observed for TcY-L2^−/−^ parasites, which were the less infective ones. As expected, for both host cells, the number of parasites per infected cell was about one, for all tested groups.

### *T. cruzi* Adhesion Profile Is Also Affected by Residence in a LAMP-Deficient Intracellular Environment, but Does Not Correlate With Parasite Invasion Rate

*T. cruzi* adhesion to the host cell has been considered as the initial step for the invasion process (Andrews and Colli, 1982; Piras et al., 1982; Piras et al., 1983), since this proximity could aid the interaction between the parasite and host cell surface proteins, which is responsible for triggering the necessary signaling pathways that lead to parasite internalization (Fernandes and Andrews, 2012). To investigate whether the differences found in the parasite invasion rates were related to their ability in adhering to these cells, we exposed pre-fixed L6 myoblasts or WT fibroblasts to TcY-WT, TcY-L1/2^−/−^, and TcY-L2^−/−^ trypomastigotes at an MOI of 50, for 40 min, and analyzed parasites that remained attached to the cell surface after several washes.

The adhesion assay in L6 myoblasts, revealed that TcY-L1/2^−/−^ trypomastigotes presented the highest adhesion rates when compared to TcY-WT and TcY-L2^−/−^ parasites (**Figure 1C**). TcY-WT parasites showed an intermediate adhesion rate compared to TcY-L2^−/−^ parasites, which in turn showed the lowest adhesion rates (**Figure 1C**). For WT fibroblasts, TcY-L1/2^−/−^ trypomastigotes presented the lowest adhesion rates when compared to TcY-WT and TcY-L2^−/−^ parasites (**Figure 1D**). There was no statistically significant difference between TcY-L2^−/−^ and TcY-WT parasites. Despite the differences found in the adhesion rates among the studied parasites in the two studied cell types, these results revealed that adhesion and invasion rates did not necessarily correlate with each other.

### *T. cruzi* Biological Characteristics Correlates to its Invasion Profile Reinforcing the Modulatory Role of Residing in a LAMP-Deficient Intracellular Environment

**Figure 2:**
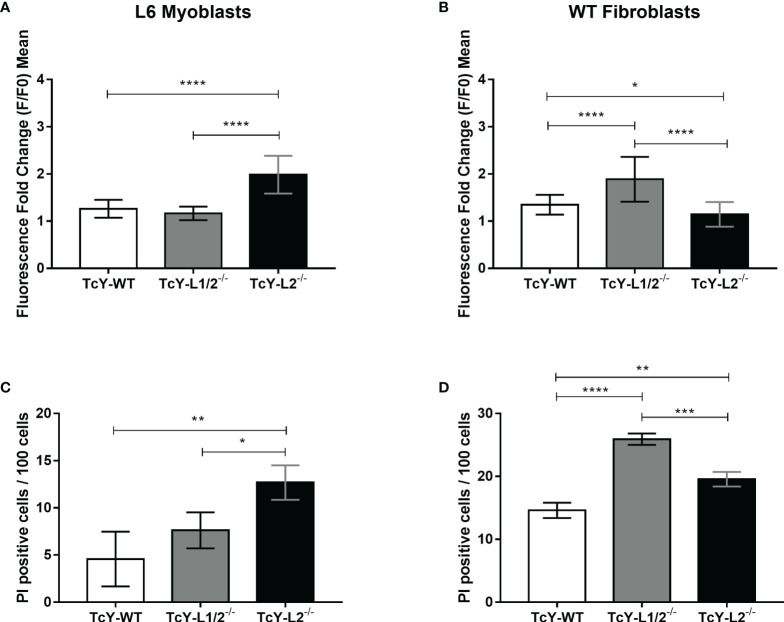
Parasites derived from WT or LAMP deficient cells differ in their ability in triggering host calcium signaling events, and these differences correlate, in part, with their ability to inducing microinjuries in the host cell plasma membrane. L6 myoblasts or WT fibroblasts were exposed to TcY-WT, TcY-L1/2^−/−^, and TcY-L2^−/−^ trypomastigotes as described in *Materials and methods*. Calcium signaling assays in L6 myoblasts **(A)** and WT fibroblasts **(B)**; the cells were also exposed to ionomicin as positive control and culture medium as negative control. Membrane injury assays in L6 myoblasts **(C)** and WT fibroblasts **(D)**. Asterisks represent statistical difference between groups (*p < 0.05, **p < 0.01, ***p < 0.001, ****p < 0.0001, one-way ANOVA, Fisher’s LSD).

As mentioned before, *T. cruzi* invasion into host cells is dependent on the parasite’s ability to trigger host cell intracellular calcium signaling and subverting the host cell plasma membrane repair mechanism. *T. cruzi* is able to initiate this calcium signaling by both causing microlesions in the host cell plasma membrane or inducing calcium signaling events through engagement of host cell surface receptors (Fernandes and Andrews, 2012). The increase in intracellular calcium concentration leads to lysosomal exocytosis and compensatory endocytosis events, culminating in parasite internalization (Tardieux et al., 1994). Since calcium ions play a crucial role in *T. cruzi* invasion, we decided to investigate whether the changes observed for the invasion rates could correlate to parasite ability in triggering calcium signals. For that, L6 myoblasts or WT fibroblasts were loaded with the fluorescent calcium-binding probe Fluo4-AM and exposed to TcY-WT, TcY-L1/2^−/−^, and TcY-L2^−/−^ infective trypomastigote forms at an MOI of 100, while recording the fluorescence increase in real time by confocal microscopy. For L6 myoblasts, calcium signaling induced by TcY-L2^−/−^ was significantly higher than the signaling induced by TcY-WT or TcY-L1/2^−/−^ parasites, while those two did not present statistically significant differences between them (**Figure 2A**, **Supplementary Figure S1**). On the other hand, for WT fibroblasts, calcium signaling induced by TcY-L2^−/−^ parasites was significantly lower than the signaling induced by the other two parasites, TcY-WT and TcY-L1/2^−/−^, being the highest calcium signals induced by TcY-L1/2^−/−^ parasites (**Figure 2B**, **Supplementary Figure S2**). For both host cell types, myoblasts and fibroblasts, the calcium signals induced by the different parasites directly correlated with their invasion rates, meaning the higher the calcium signaling triggered by the parasite, the higher the cell invasion efficiency. These results reinforce the important role played by calcium signaling events during the *T. cruzi* invasion process.

To investigate further the differences in the calcium signaling triggered by the distinct parasites in the different cell types, we evaluated whether the calcium-signaling events were generated by micro-injuries at the host cell plasma membrane or triggered by activation of host cell surface receptors. To investigate the calcium signaling induced by plasma membrane injuries, we exposed L6 myoblasts or WT fibroblasts to TcY-WT, TcY-L1/2^−/−^, and TcY-L2^−/−^ trypomastigotes at an MOI of 100 in the presence of propidium iodide (PI) for 30 min. PI is a red-fluorescent nuclear and chromosome counterstain, not permeant to live cells. Therefore, PI staining indicates membrane permeabilization by injury. For L6 myoblasts, TcY-L2^−/−^ parasites induced injury to a higher number of cells when compared to TcY-WT and TcY-L1/2^−/−^ parasites, corroborating their invasion profile and suggesting that for these parasites, calcium signals are triggered through injuries at the host cell membrane (**Figure 2C**). No statistically significant differences were observed between the number of PI positive cells for TcY-WT and TcY-L1/2^−/−^ parasites, indicating that these parasites likely induce similar levels of injury to L6 myoblasts (**Figure 2C**, **Supplementary Figure S3**). Again, these results corroborate with the invasion profile observed for these parasites. Alternatively, cells were incubated with parasites in the absence of PI, washed, and then incubated with PI. As expected, no statistically significant differences were observed among PI staining of cells incubated with PI post *T. cruzi* infection, for all parasites, and the negative control (cells incubated with PI only, without being previously exposed to the parasite) (**Supplementary Figure S4**). For WT fibroblasts, TcY-L1/2^−/−^ parasites induced injury to a higher number of cells when compared to TcY-WT and TcY-L2^−/−^ parasites, corroborating their invasion profile in these cells (**Figure 2D** and **Supplementary Figure S5**). TcY-WT parasites induced injury to the lowest number of cells, when compared to the others, even though calcium signaling triggered by these parasites in WT fibroblasts was higher than that observed for TcY-L2^−/−^ parasites (**Figures 2B, D**).

### *T. cruzi* Proteome Is Influenced by the Residence in a LAMP-Deficient Intracellular Environment

Once parasites released from the distinct cell types presented different biological behaviors, we decided to investigate whether changes in these parasites’ surface membrane protein profiles could account for the biological phenotypes obtained. For this, we performed quantitative mass spectrometry-based proteomic analysis to identify and quantify the membrane-enriched protein preparation from TcY-WT, TcY-L1/2^−/−/−^, and TcY-L2^−/−^ trypomastigotes. *T. cruzi* Y strain trypomastigotes were cultured in the wild-type or LAMP-deficient fibroblasts, collected, lysed, and then ultra-centrifuged to separate the membrane-enriched protein fraction. Extracted proteins were digested, reduced, and alkylated, and the tryptic peptides were labeled with TMT 6-plex (Thermo Fisher), analyzed by mass spectrometry-based proteomics, and submitted to bioinformatics tools to perform functional analyses. For protein identification from the peptide sequences, we used the Y strain annotated proteins from TriTrypDB website (released on April 23, 2020 and downloaded on May 2020) (Callejas-Hernandez et al., 2018).

We identified 3,806 proteins with high confidence level and containing at least one unique peptide in all three samples (**Supplementary Table S1**). A principal component analysis was performed to evaluate the biological replicates similarity, using the normalized quantitative abundance values (**Supplementary Figure S6**). This analysis showed an acceptable level of similarity between replicates within the same group. A total of 1,365 proteins were annotated as uncharacterized or hypothetical proteins, corresponding to about 36% of the identified proteins in our data set. The high percentage of uncharacterized proteins is not surprising, since for the *T. cruzi* Y strain genome, 56.94% of the proteins correspond to hypothetical proteins of unknown function (Callejas-Hernandez et al., 2018). Because of this, we performed a GO analysis with Fisher exact test filtering for FDR <0.05, using the GO enrichment analysis tool on the TriTrypDB in order to verify if our samples were in fact enriched in membrane terms. Regarding the GO cellular component level, the analysis showed that our data set presented an enrichment in many terms related to membrane, such as intrinsic component of membrane, integral component of membrane, and endomembrane system (**Supplementary Figure S7**). Even though the most enriched term is cytoplasm, the QuickGO definition of this term comprises the contents of a cell excluding the plasma membrane and nucleus, but including other subcellular structures (https://www.ebi.ac.uk/QuickGO/GTerm?id=GO:0005737). Therefore, many organelle membrane proteins present in our data set fell into this GO cytoplasm term.

We then compared the proteomes of TcY-L1/2^−/−^ and TcY-L2^−/−^ trypomastigotes’ membrane-enriched proteins preparation to that from TcY-WT trypomastigotes. We considered as regulated proteins, all members that presented a log2 fold change higher than the mean plus 2 times the standard deviation or lower than the mean minus 2 times the standard deviation and coefficient of variation lower than 40%—referred from here as the regulated subproteome. We found 75 and 90 proteins in the TcY-L1/2^−/−^ and TcY-L2^−/−^ regulated subproteomes, respectively (**Supplementary Table S2**) that matched these criteria. From these proteins, either a considerable percentage, in both regulated subproteomes, had predicted transmembrane domains or to be classically or non-classically secreted (**Table 1**). Additionally, important *T. cruzi* virulence factors, such as members of trans-sialidase superfamily, and other surface proteins families, such as MASP and surface protease gp63 (Goncalves et al., 1991; Trocoli Torrecilhas et al., 2009; Bayer-Santos et al., 2013), were found to be differentially regulated in these parasites (**Table 2**).

**Table 1:** Number of proteins from the regulated subproteome with predicted transmembrane domains, GPI-anchor, classically and non-classically secreted, and enzymes.

Subproteome	Total regulated	TM Domains*	GPI anchored*	SignalP*	SecretomeP*	Enzymes**
TcY-L1/2^−/−^	75	27 (36.0%)	8 (10.7%)	7 (9.3%)	19 (25.3%)	20 (26.7%)
↑ UP	51	26 (51.0%)	8 (15.7%)	6 (11.8%)	13 (25.5%)	12 (23.5%)
↓ DOWN	24	1 (4.2%)	0 (0.0%)	1 (4.2%)	4 (16.7%)	6 (25.0%)
TcY-L2^−/−^	90	34 (37.8%)	8 (8.9%)	16 (17.8%)	11 (12.2%)	23 (25.6%)
↑ UP	55	31 (56.4%)	7 (12.7%)	12 (21.8%)	5 (9.1%)	18 (32.7%)
↓ DOWN	35	3 (8.6%)	1 (2.9%)	4 (11.4%)	5 (14.3%)	5 (14.3%)

*Absolute numbers and percentages of regulated protein groups with predicted transmembrane (TM) domains or GPI-anchored, ER/Golgi signal peptide (SignalP), or non-classically secreted (SecretomeP) related to the total number of regulated proteins identified in each subproteome compared to TcY-WT proteome.

**Absolute numbers and percentages of enzymes in each subproteome related to the total number of regulated proteins compared to TcY-WT proteome.

**Table 2:** Important surface membrane proteins regulated in TcY-L1/2^−/−^ and TcY-L2^−/−^ trypomastigotes compared to TcY-WT trypomastigotes.

TriTryp ID	Protein description	log2 FC	log2 FC
		TcY-L1/2^−/−^/TcY-WT	TcY-L2^−/−^/TcY-WT
TcYC6_0114230	membrane protein, putative	NDR	1.04
TcYC6_0162290	Mucin-associated surface protein (MASP), subgroup S002	NDR	0.88
TcYC6_0158360	Mucin-associated surface protein (MASP), subgroup S017	0.88	NDR
TcYC6_0161780	Mucin-associated surface protein (MASP), subgroup S061	NDR	−1.66
TcYC6_0160330	Mucin-associated surface protein (MASP), subgroup S097	0.31	NDR
TcYC6_0124770	Surface membrane protein	NDR	1.08
TcYC6_0169700	Surface protease GP63, putative	NDR	1.32
TcYC6_0169840	Surface protease GP63, putative	0.29	NDR
TcYC6_0016150	Transmembrane protein	NDR	−0.98
TcYC6_0128720	Trans-sialidase, Group II, putative	0.8	NDR
TcYC6_0131410	Trans-sialidase, Group II, putative	0.36	NDR
TcYC6_0131440	Trans-sialidase, Group II, putative	NDR	0.87
TcYC6_0130520	Trans-sialidase, Group V, putative	0.69	NDR
TcYC6_0129770	Trans-sialidase, Group VI, putative	0.32	NDR
TcYC6_0128910	Trans-sialidase, putative	0.78	NDR
TcYC6_0130010	Trans-sialidase, putative	NDR	0.9

NDR, not differentially regulated; red, upregulated; blue, downregulated

**Figure 3:**
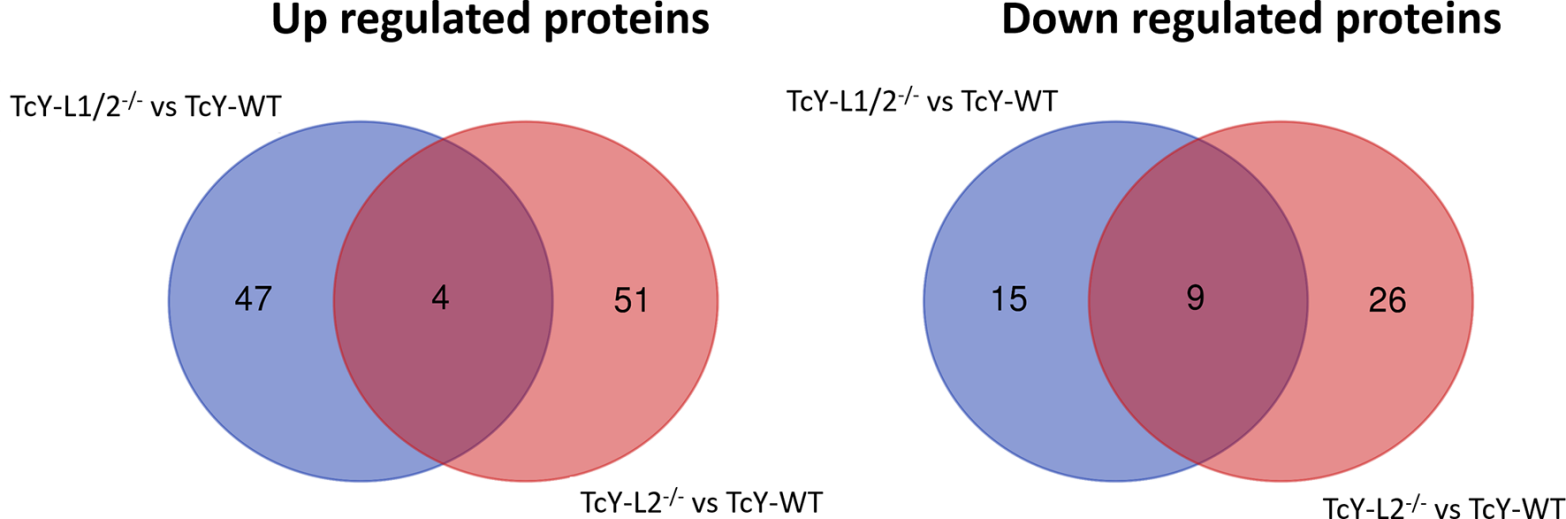
TcY-WT, TcY-L1/2^−/−^, and TcY-L2^−/−^ trypomastigotes present differentially regulated proteins among them. Venn diagrams showing the exclusively regulated and the shared proteins in TcY-L1/2^−/−^ and TcY-L2^−/−^ regulated subproteomes in comparison to TcY-WT proteome. This figure was prepared using the Draw Venn Diagram webtool (http://bioinformatics.psb.ugent.be/webtools/Venn/).

In the TcY-L1/2^−/−^ regulated subproteome, 51 proteins were upregulated and 24 were downregulated, and in the TcY-L2^−/−^ regulated subproteome, 55 proteins were upregulated and 35 were down regulated, when compared to TcY-WT proteome. There were only four upregulated proteins, three of them being hypothetical proteins, and nine downregulated proteins shared between TcY-L1/2^−/−^ and TcY-L2^−/−^ regulated subproteomes (**Figure 3**).

**Figure 4:**
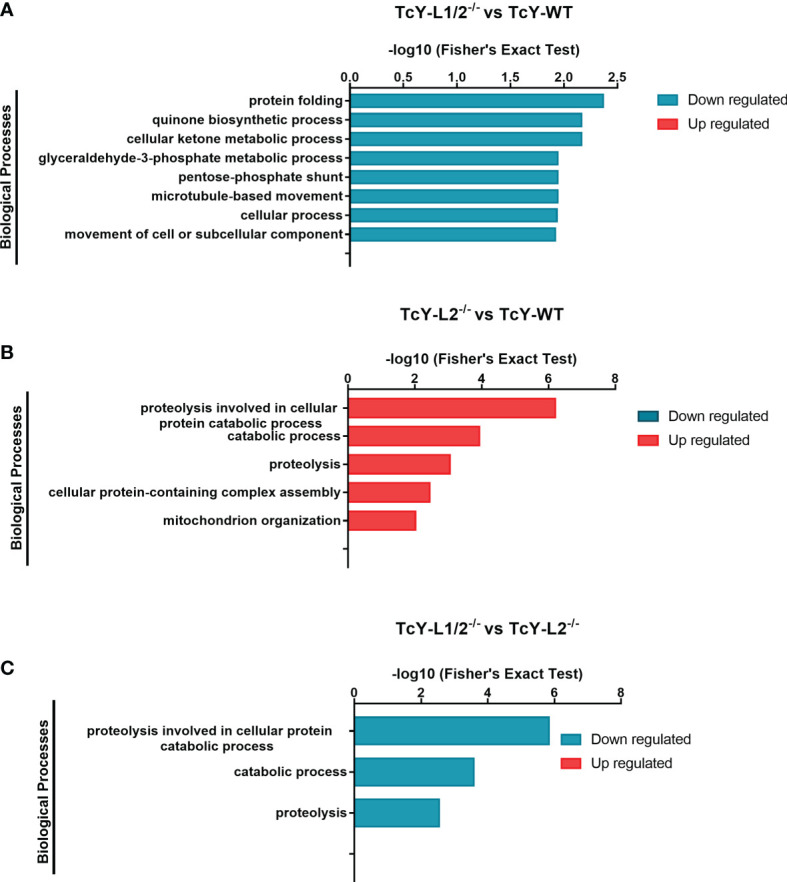
Gene ontology enrichment analysis of biological processes terms in TcY-L1/2^−/−^- or TcY-L2^−/−^-regulated subproteomes. The graph shows the different biological processes up- or downregulated, based on the differentially expressed proteins of TcY-L1/2^−/−^ **(A)** or TcY-L2^−/−^ **(B)** compared to TcY-WT proteome and on the differentially expressed proteins of TcY-L1/2^−/−^ **(C)** compared to TcY-L2^−/−^ proteome.

For further understanding the possible role of these differentially regulated proteins in the biological behavior observed for TcY-L1/2^−/−^ and TcY-L2^−/−^, we classified and grouped them in distinct biological processes, based on their gene ontology, using the enrichment test available on TriTrypDB with Fisher exact test filtering for FDR <0.05. Regarding the TcYL1/2−/− regulated subproteome regulated subproteome, there was no enriched biological processes (BP) from the up regulated proteins and we found 15 BP enriched from the down regulated. We removed the redundant terms from them using the REVIGO tool (http://revigo.irb.hr/), and after summarizing the terms, we found eight BP downregulated (protein folding, cellular ketone metabolic process, quinone biosynthetic process, pentose-phosphate shunt, glyceraldehyde-3-phosphate metabolic process, microtubule-based movement, cellular process, movement of cell or subcellular component) in comparison to TcY-WT proteome (**Figure 4A** and **Supplementary Table S3**). Six proteins were relevant for two or more downregulated BP in theTcY-L1/2^−/−^ regulated subproteome: 6-phosphogluconate dehydrogenase, decarboxylating, putative (TcYC6_0069460), ubiquinone biosynthesis protein COQ7-like, putative (TcYC6_0110760), kinesin-13 3, putative (TcYC6_0081520), kinesin, putative (TcYC6_0110780), T-complex protein 1, delta subunit, putative (TcYC6_0023210), and chaperonin/T-complex protein 1 gamma subunit, putative (TcYC6_0030620). We next performed a GO biological process enrichment analysis using the membrane-associated proteins that presented transmembrane domains and/or were assigned as membrane by the DeepLoc analysis (referred from here as membrane subproteome). From this analysis, we did not find any enriched BP either from the up- or the downregulated proteins list. However, for the upregulated membrane subproteome, we found the sphingolipid metabolism (ec00600:PK:KEGG) pathway enriched, using the Metabolic Pathway Enrichment analysis tool on TriTrypDB with KEGG as pathway source and filtering FDR <0.05.

For TcY-L2^−/−^ regulated subproteome, there was no enriched BP from the downregulated proteins, and we found 25 BP enriched from the upregulated. After summarizing the terms with the REVIGO tool, we found five BP upregulated (proteolysis involved in cellular protein catabolic process, catabolic process, proteolysis, cellular protein-containing complex assembly, mitochondrion organization) in comparison to TcY-WT proteome (**Figure 4B** and **Supplementary Table S3**). Six proteins were present in two or more upregulated BP: proteasome alpha 1 subunit, putative (TcYC6_0071370), proteasome alpha 2 subunit, putative (TcYC6_0023180), proteasome beta 3 subunit, putative (TcYC6_0044600), proteasome beta 6 subunit, putative (TcYC6_0041700), proteasome beta 7 subunit, putative (TcYC6_0091650), and putative mitochondrial chaperone BCS1 (TcYC6_0123290). Using the Metabolic Pathway Enrichment analysis tool on TriTrypDB with KEGG as pathway source and filtering FDR <0.05, we found two upregulated pathways [arginine and proline metabolism (ec00330:PK:KEGG), and Diterpenoid biosynthesis (ec00904:PK:KEGG)] and one downregulated pathway [ether lipid metabolism (ec00565:PK:KEGG)]. The GO biological process enrichment analysis of TcY-L2^−/−^ membrane subproteome revealed one upregulated BP, the vacuolar proton-transporting V-type ATPase complex assembly. We did not find any enriched pathways for this membrane subproteome.

In order to establish a better understanding of the LAMP-deficient cells-derived parasites, we compared the TcY-L1/2^−/−^ and TcY-L2^−/−^ proteomes. These parasites were the most different regarding their invasion characteristics. In fact, the proteomic analysis revealed a higher number of differentially regulated proteins between them, 136 proteins—55 downregulated and 81 upregulated (using the same previously described criteria) (**Supplementary Table S2**). From these proteins, a considerable part is predicted to have transmembrane domains or to be classically or non-classically secreted (**Table 3**). Interestingly, we also found a higher number of membrane surface proteins regulated between TcY-L1/2^−/−^ and TcY-L2^−/−^ trypomastigotes (**Table 4**).

**Table 3:** Number of proteins from the regulated subproteome with predicted transmembrane domains, GPI-anchor, classically and non-classically secreted, and enzymes.

Subproteome	Total regulated	TM Domains*	GPI anchored*	SignalP*	SecretomeP*	Enzymes**
TcY-L1/2^−/−^ vs TcY-L2^−/−^	136	34 (25.0%)	10 (7.4%)	18 (13.2%)	28 (20.6%)	39 (28.7%)
↑ UP	81	10 (12.3%)	2 (2.5%)	6 (7.4%)	17 (21.0%)	17 (21.0%)
↓ DOWN	55	24 (43.6%)	8 (14.5%)	12 (21.8%)	11 (20.0%)	22 (40.0%)

*Absolute numbers and percentages of regulated protein groups with predicted transmembrane (TM) domains or GPI-anchored, ER/Golgi signal peptide (SignalP), or non-classically secreted (SecretomeP) related to the total number of regulated proteins identified in each subproteome compared to TcY-WT proteome.

**Absolute numbers and percentages of enzymes in each subproteome related to the total number of regulated proteins compared to TcY-WT proteome.

**Table 4:** Important surface membrane proteins regulated in TcY-L1/2^−/−^ trypomastigotes compared to TcY-L2^−/−^ trypomastigotes.

TriTryp ID	Protein description	log2 FC
		TcY-L1/2^−/−^/TcY-L2^−/−^
TcYC6_0154080	mucin TcMUCII, putative	−1.46
TcYC6_0150670	mucin TcMUCII, putative	−0.98
TcYC6_0154140	mucin TcMUCII, putative	0.38
TcYC6_0158360	Mucin-associated surface protein (MASP), subgroup S017	0.71
TcYC6_0164190	Mucin-associated surface protein (MASP), subgroup S054	−0.98
TcYC6_0161500	Mucin-associated surface protein (MASP), subgroup S074	−1.47
TcYC6_0124770	Surface membrane protein	−1.09
TcYC6_0169090	Surface protease GP63, putative	−1.11
TcYC6_0077110	Surface protein TolT, putative	−1.5
TcYC6_0078130	Surface protein TolT, putative	−1.46
TcYC6_0078140	Surface protein TolT, putative	−1.44
TcYC6_0141330	Trans-sialidase (pseudogene), putative	0.34
TcYC6_0130150	Trans-sialidase, Group II, putative	−1.18
TcYC6_0131910	Trans-sialidase, Group II, putative	−1.01
TcYC6_0132210	Trans-sialidase, Group II, putative	−1.01
TcYC6_0128720	Trans-sialidase, Group II, putative	0.88
TcYC6_0130010	Trans-sialidase, putative	−0.93
TcYC6_0128910	Trans-sialidase, putative	0.76

Red, upregulated; blue or purplish, downregulated.

The GO BP enrichment analysis from these differentially regulated proteins between TcY-L1/2^−/−^ and TcY-L2^−/−^ trypomastigotes showed 13 BPs from the down regulated proteins list and no BP from the upregulated protein list. After summarizing the terms using the REVIGO tool, three downregulated BP remained (proteolysis involved in cellular protein catabolic process, catabolic process, and proteolysis) (**Figure 4C** and **Supplementary Table S3**). Five proteasome proteins were present in all three BPs: proteasome alpha 1 subunit, putative (TcYC6_0071370), proteasome alpha 2 subunit, putative (TcYC6_0023180), proteasome alpha 7 subunit, putative (TcYC6_0044220), proteasome beta 3 subunit, putative (TcYC6_0044600), and proteasome beta 7 subunit, putative (TcYC6_0091650). The metallo-peptidase, Clan MF, Family M17, putative (TcYC6_0033380), cytoskeleton-associated protein CAP5.5, putative (TcYC6_0107360), and surface protease GP63, putative (TcYC6_0169090) proteins also contributed to the proteolysis enrichment.

### Residence in a LAMP-2-Deficient Intracellular Environment Increases *T. cruzi* Proteolytic Activity

**Figure 5:**
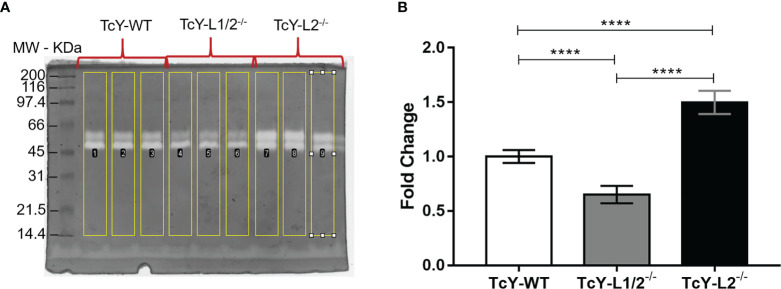
Increased proteolytic activity of TcY-L2^−/−^ assessed by gelatin zymography assays. **(A)** Representative SDS-PAGE gel showing the negative bands from gelatinolytic activity. **(B)** Quantification obtained from gels using ImageJ software—TcY-WT values were used as a normalizer for all groups. Asterisks represent statistical difference between groups (****p < 0.0001, one-way ANOVA, Fisher’s LSD).

Since many proteolysis-related terms were enriched in the TcY-L2^−/−^ regulated subproteome compared to TcY-WT and TcY-L1/2^−/−^ proteomes, we decided to further investigate the parasites proteolytic activity. For that, we evaluated the parasites’ proteolytic activity through gelatin zymography assays. The results showed a higher gelatinolytic activity for TcY-L2^−/−^ trypomastigotes compared to TcY-WT and TcY-L1/2^−/−^ (**Figure 5**), thus corroborating the proteomic analysis.

**Figure 6:**
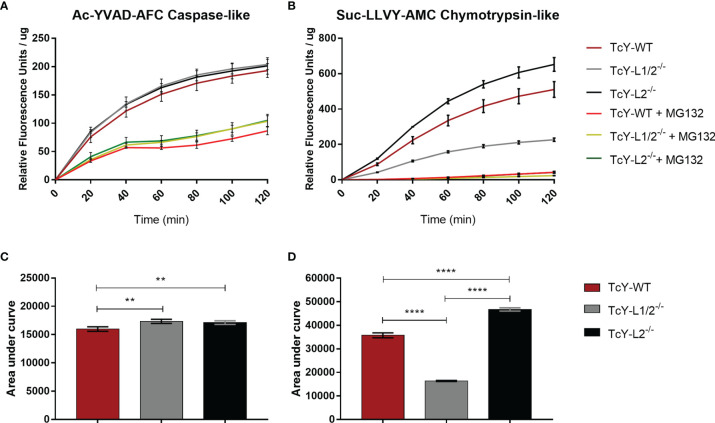
Cysteine protease and serine protease enzymatic activity kinetics. Cysteine **(A)** or serine **(B)** activity in TcY-WT, TcY-L1/2^−/−^, or TcY-L2^−/−^ trypomastigotes protein extracts were assayed using a fluorescence method with caspase substrate Ac-YVAD-AFC for caspase-like cysteine enzyme activity or chymotrypsin substrate Suc-LLVY-AMC for chymotrypsin-like serine enzyme activity. The data are mean ± SD for the kinetic curves **(A, B)** (**p < 0.01, ****p < 0.0001, one-way ANOVA, Fisher’s LSD).

In order to investigate deeper the higher proteolytic profile observed for TcY-L2^−/−^ trypomastigotes, we decided to test the protein extracts for serine proteases and cysteine proteases activity. Enzyme activity assays were performed using Ac-YVAD-AFC caspase-like and Suc-LLVY-AMC chymotrypsin-like fluorogenic substrates, for cysteine and serine proteases, respectively. For caspase-like activity, TcY-L1/2^−/−^ and TcY-L2^−/−^ protein extracts showed slightly higher activities than TcY-WT protein extract (**Figures 6A, C**). For all protein extracts, the inhibitor MG-132 decreased caspase-like activity in 2.23 ± 0.055, 1.98 ± 0.25, and 1.94 ± 0.34 times for TcY-WT, TcY-L1/2^−/−^, and TcY-L2^−/−^, respectively (**Figures 6A, C**). For chymotrypsin-like activity, TcY-L2^−/−^ protein extract showed the highest activity, while TcY-L1/2^−/−^ presented the lowest (**Figures 6B, D**). Here again, for all protein extracts, the inhibitor MG-132 considerably decreased their activities in 12.12 ± 1.53, 8.91 ± 0.48, and 15.73 ± 0.53 times for TcY-WT, TcY-L1/2^−/−^, and TcY-L2^−/−,^ respectively. For Suc-LLVY-AMC substrate, the kinetic activity also showed a similar profile to the one revealed by the zymography assays.

## Discussion

In previous studies carried out by our group, we showed that the alteration of a single protein in the host cell—LAMP (a lysosomal protein)—changed the biological behavior of two distinct *T. cruzi* strains, Y and CL, belonging to TcI and TcVI lineages, respectively (Albertti et al., 2010). It was shown that parasites from both strains increased their intracellular growth in LAMP-deficient cells (LAMP1/2^−/−^), releasing a significantly higher number of trypomastigote forms at the end of a single intracellular cycle, when compared to infections on wild-type (WT) cells (Albertti et al., 2010). Lysosomes are relevant host-cell organelles that affect *T. cruzi* invasion success, since they are recruited to the parasite entry site, not only donating membrane for the formation of the parasitophorous vacuole (Tardieux et al., 1992) but also are crucial for parasite’s permanence in the cell (Andrade and Andrews, 2004). Therefore, the present study aimed to understand whether the parasites released from the different intracellular environments, therefore from the LAMP-deficient or WT cells, exhibited different biological behavior among them. For this, we evaluated four characteristics related to the parasite’s entry process into the host cell: the invasion and adhesion rates, the calcium signal profile induced by these parasites, and their ability to induce host cell membrane micro-injuries. These parameters were evaluated in two different host cell lines: L6 myoblasts and WT fibroblasts.

Our results first revealed that only one passage through a LAMP-deficient environment was able to modify the parasite’s invasion rate. To the best of our knowledge, this is the first time that it is shown that one single passage through different intracellular environments may induce such significant changes in the parasite behavior. As mentioned before, in the work from Dos Santos et al. (2012), changes in the MASP expression profile or invasion rates were studied upon several passages through different cell types (dos Santos et al., 2012). Additionally, we showed that the absence of only LAMP-2 or both LAMP proteins affect the parasite differently. Although LAMP2^−/−^ and LAMP1/2^−/−^ cells have similar lysosomal distribution and enzyme activity, it has been shown that the latter present an increased number of autophagic vacuoles, both before or after starvation, in relation to LAMP2^−/−^ or WT cells (Eskelinen et al., 2004; Eskelinen, 2006). Additionally, the level of cholesterol accumulation in endo-/lysosomal compartments is distinct for *LAMP2* ^−/−^ and *LAMP1/2* ^−/−^ cells (Tanaka et al., 2000; Eskelinen et al., 2002; Eskelinen et al., 2004). In *LAMP1/2* ^−/−^ cells, due to this disturbed cholesterol traffic, there was also a decrease in amount of lipid droplets (Eskelinen et al., 2004). Apparently, the lack of both LAMPs implicates in an exacerbated phenotype. In fact, LAMP-2-deficient mice complete its embryonic development, although 50% die shortly after they are born (between 20 and 40 postnatal day), while the deficiency in both LAMP-1 and 2 is embryonic lethal with prenatal death between embryonic days 14.5 and 16.5. The above-mentioned indicates that the intracellular environment and cellular behavior are not similar between LAMP2^−/−^ and LAMP1/2^−/−^ cells. These changes may likely signal differently to intracellular *T. cruzi*. Autophagosomes have been shown to participate in *T. cruzi* infection in host cells (Romano et al., 2009). In this study, Romano et al. (2009) showed that the induction of autophagy favors parasite invasion and that an increasing number of internalized parasites colocalize with LC3, an autophagosome marker, with time of infection (Romano et al., 2009). Whether the percentage of parasites colocalizing with autophagosome markers or even the time length of association of these markers varies among WT, LAMP2^−/−^, and LAMP1/2^−/−^ cells is not known, but if so, they may account for at least part of the distinct behavior observed for the parasites release from LAMP2^−/−^ and LAMP1/2^−/−^ cells. We also showed that the cell type that the parasite is interacting with affected the invasion rate. For example, we observed that the TcY-L1/2^−/−^ trypomastigotes were the most infective in WT fibroblasts but the least infective in L6 myoblasts. On the other hand, TcY-L2^−/−^ trypomastigotes were the least infective in WT fibroblasts, but the most infective in L6 myoblasts. In fact, differences in *T. cruzi*’s ability to invade different cell types has been shown previously (Andrade et al., 2010; Maeda et al., 2016). This has to do with the fact that although parasite invasion is always dependent on calcium signaling, lysosome recruitment, and fusion, the pathways involved in eliciting these signals may vary a lot among different parasite strains and the host cell type (Burleigh and Woolsey, 2002; Barrias et al., 2013; Ferri and Edreira, 2021).

The recruitment and fusion of lysosomes, crucial steps for the invasion process, occur at the parasite adhesion site (Tardieux et al., 1992; Tardieux et al., 1994), and the existence of a correlation between cell adhesion and invasion rates by *T. cruzi* has been reported (Piras et al., 1982; Andrews and Colli, 1982; Piras et al., 1983). Therefore, we also investigated whether the adhesion profiles of TcY-WT, TcY-L1/2^−/−^, or TcY-L2^−/−^ trypomastigotes differed from each other. Indeed, the adhesion profiles not only varied among the different parasites but also between the two cell types for the same parasite population. Data obtained here, from both invasion and adhesion rates, demonstrate that developing in an intracellular environment absent in LAMP proteins most likely induces changes in the expression of trypomastigote surface proteins. It also reinforces data from the literature demonstrating that changes in the *T. cruzi*’s trypomastigotes expression profile alters parasite tropism for specific cells (Andrade et al., 1999; Macedo et al., 2004; dos Santos et al., 2012). Nonetheless, contrary to expected, no correlation between adhesion and invasion was observed. In fact, we observed an inverse relation for some cell types, since less adherent parasites had the highest invasion rates. Again, to the best of our knowledge, this is the first time that an opposite relation between adhesion and invasion rates are shown for *T. cruzi*. These data also support the idea that proteins involved in adhesion may differ from those required for signaling and triggering the cell invasion process, the latter being probably the main factor in the host cell infection process. Therefore, a high adhesion with a low cell signaling would be ineffective to increase invasion.

Based on the above-mentioned, we investigated whether differences in the invasion rates among TcY-WT, TcY-L1/2^−/−^, or TcY-L2^−/−^ would correlate with these parasites’ ability to trigger host intracellular calcium signaling. As mentioned previously, calcium signaling, either induced *via* surface receptors stimulus and intracellular calcium stock mobilization or *via* extracellular calcium influx triggered by microinjuries, is a crucial step to *T. cruzi* invasion success (Fernandes and Andrews, 2012). Indeed, a direct correlation between the amount of calcium signaling and invasion rates was observed, corroborating the data observed for cell invasion. To understand further the contribution of calcium triggered *via* host surface receptor engagement and parasite induced host membrane microinjuries, we also performed a membrane injury assay. For most studied parasites and cell types tested, calcium signaling correlated with parasites’ ability to induce host cell membrane microinjuries, showing that this pathway is relevant for parasite host cell entry. However, for TcY-WT and TcY-L2^−/−^ in WT fibroblasts, this correlation was not observed. TcY-WT showed significantly higher invasion rates and calcium signals in WT fibroblasts when compared to TcY-L2^−/−^ trypomastigotes, although lower levels of host cell membrane microinjury when compared to TcY-L2^−/−^. Thus, calcium from the extracellular milieu would not be enough to account for the results obtained, meaning that calcium from intracellular stocks, released from the ER *via* host cell surface protein signaling, triggered by the parasite, was likely to contribute. These results reinforce that both calcium signaling *via* microinjury and *via* host cell membrane receptors are relevant to *T. cruzi*’s invasion process. Additionally, these results reinforce that, without the ability of parasites in successfully triggering calcium signaling events in the host cell, adhesion alone is not enough for inducing parasite invasion. In fact, proteins involved in *T. cruzi* adhesion (members of trans-sialidase or MASPS families for example) to host cells are usually not the ones shown to induce calcium signaling (calcium agonist generated by oligopeptidase B from the parasite or bradykinin obtained from the activity of cruzipain from the parasite) (Burleigh and Woolsey, 2002; Alves and Colli, 2007).

All observed biological alterations for all three parasites groups indicated differences in their surface proteins expression profile. Based on our data, we hypothesize that the intracellular environment can induce changes in the parasite during its development, including surface molecules. To analyze possible differences in the protein surface profiles among the three studied parasites, we performed a quantitative mass-spectrometry-based proteomic analysis in a membrane-enriched protein preparation.

The data obtained from this approach confirmed that the intracellular environment is indeed able to modulate *T. cruzi*’s protein abundance. In comparison to TcY-WT trypomastigotes, TcY-L1/2^−/−^ and TcY-L2^−/−^ regulated subproteomes only share 13 regulated proteins (four upregulated and nine downregulated—**Figure 4**), which confirms that only one cycle of infection in a different intracellular environment can induce changes in the parasite surface. Additionally, we observed that a considerable percentage of proteins had predicted transmembrane domains or to be classically or non-classically secreted (**Tables 1**, **3**). These percentages are similar to previously published data from tissue-culture-derived trypomastigotes and axenic amastigotes cell surface proteomes (Queiroz et al., 2014) and *T. cruzi*’s secretome (Bayer-Santos et al., 2013).

From the comparison between the TcY-L1/2^−/−^ or TcY-L2^−/−^ and the TcY-WT proteome (**Table 2**) and between the TcY-L1/2^−/−^ and the TcY-L2^−/−^ proteome (**Table 4**), several glycosylphosphatidylinositol (GPI)-anchored proteins, such as trans-sialidases, mucins, MASP members, and gp63 proteins, were found regulated. It is very well known in the literature that GPI-anchored proteins widely coat *T. cruzi*’s plasma membrane and are involved in several aspects of host–parasite interactions, such as adhesion to and invasion of host cells, evasion from the host immune system, and pathogenesis (McConville and Ferguson, 1993; Ferguson, 1999; McConville and Menon, 2000; Buscaglia et al., 2006; Gazzinelli and Denkers, 2006). Mucin and trans-sialidase protein families are very relevant to *T. cruzi*’s invasion process (McConville and Menon, 2000). The latter protein family can bind to host cell receptors and transfer sialic-acid residues from host glycoconjugates to the major surface glycoproteins of *T. cruzi*—the mucin family, which has been shown to be important for parasite host cell invasion (Buscaglia et al., 2006). Antibodies raised against synthetic glycopeptides that mimic the parasite mucin glycoproteins remarkably affected *T. cruzi* Y strain metacyclic trypomastigotes invasion in LLC-MK2 cells after incubation (Campo et al., 2014). Another work showed that purified GPI mucins from *T. cruzi* modulated trypomastigote invasion during interaction with LLC-MK2 cells (Soares et al., 2012). A transcriptomic analysis of a virulent (CL Brener) and a non-virulent (CL-14) strain from *T. cruzi* revealed different patterns of expression for the gene families that encode surface proteins such as trans-sialidases, mucins and MASPs (Belew et al., 2017). This comparative analysis indicated that the non-virulent phenotype of the CL-14 strain could be due, in part, to a reduced or delayed expression of genes encoding these surface proteins (Belew et al., 2017). Thus, the fact that these proteins have been shown to be differentially present in TcY-WT, TcY-L1/2^−/−^ and TcY-L2^−/−^ parasites may correlate with our biological findings, particularly with the invasion rates.

One remarkable difference observed in the protein extract from TcY-L2^−/−^ trypomastigotes was its higher levels of proteolytic activity when compared to TcY-WT and TcY-L1/2^−/−^ trypomastigotes. In fact, the GO analysis showed an enrichment in proteolysis terms for TcY-L2^−/−^ regulated subproteome, when compared to both TcY-WT and TcY-L1/2^−/−^ proteomes. Besides proteasome proteins, three other *T. cruzi* proteases were responsible for this GO enrichment: metallo-peptidase, Clan MF, Family M17, putative (TcYC6_0033380), cytoskeleton-associated protein CAP5.5, putative (TcYC6_0107360) and surface protease GP63, and putative (TcYC6_0169090). The metallo-peptidase, Clan MF, Family M17 belongs to the leucyl aminopeptidase family (EC 3.4.11.1; LAPs) and is a metalloaminopeptidase that catalyzes the removal of N-terminal amino acid residues, preferentially leucine, from proteins and peptides. This peptidase was shown to be expressed in all *T. cruzi* forms and does not present gelatinolytic activity (Cadavid-Restrepo et al., 2011). The cytoskeleton-associated protein CAP5.5 is a calpain-like protein that was first reported in *T. brucei* and presents post-translational modifications, such as myristoylation and palmytoylation, indicating an association to plasma membrane (Hertz-Fowler et al., 2001). In *T. cruzi*, the cytoskeleton-associated protein CAP5.5 was identified in both membrane and myristoylation proteomic studies (Giese et al., 2008; Kulkarni et al., 2009), and it was also present in the secretome of metacyclic trypomastigotes (Bayer-Santos et al., 2013). However, Giese et al. (2008) detected no proteolytic activity in gelatin zymography assays, which was consistent with other calpain-like proteins and suggesting that this protein could have a role in signal transduction (Giese et al., 2008). The surface protease gp63 is a very well-known protease from *T. cruzi* important for host cell infection (Kulkarni et al., 2009) and presents metalloprotease activity (Cuevas et al., 2003). In our gelatin zymography assays, we observed gelatinolytic activity that could be related to the presence of gp63, since the negative bands presented a size between 45 and 66 kDa (Rebello et al., 2019).

Other differences found upon the proteomic analysis were related to specific metabolic pathways. The pathway enrichment analysis also revealed three pathways enriched for TcY-L2^−/−^ trypomastigotes in comparison to the TcY-WT trypomastigotes (**Supplementary Table 3**), one from the down regulated proteins list (ether lipid metabolism) and two from the up regulated proteins list (arginine and proline metabolism and, diterpenoid biosynthesis). The ether lipid synthesis has not been fully studied yet and many related proteins are still to be characterized in *T. cruzi* (Booth and Smith, 2020). The amino acid proline has been demonstrated to be involved in several essential aspects of *T. cruzi*’s life cycle, such as differentiation of epimastigotes into metacyclic trypomastigotes and among intracellular stages, host cell invasion, and survival to thermal and nutritional stress (Tonelli et al., 2004; Martins et al., 2009; Paes et al., 2013). Tonelli et al. (2004) observed that under L-proline supplementation, CHO-K1-infected cell cultures released a higher number of trypomastigotes to the extracellular milieu from the fifth to the ninth day post-infection (Tonelli et al., 2004), although the authors did not measure the intracellular parasite multiplication. Based on their data, the previous data from our group (Albertti et al., 2010), and the above-mentioned pathway enrichment analysis, we are prone to believe that perhaps trypomastigotes derived from LAMP-2-deficient fibroblasts possess a more active proline metabolism, which, in turn, could also explain their higher multiplication rate previously observed by our group (Albertti et al., 2010). Experimental data are still needed to prove this hypothesis. Additionally, Martins et al. (2009) have observed a recovery of gp82-mediated invasion capacity promoted by L-proline treatment of starved *T. cruzi*’s Y strain metacyclic trypomastigotes after 54 h in starvation media (Martins et al., 2009). The authors also observed that L-proline increased *T. cruzi*’s CL strain parasites’ ability to traverse a gastric mucin layer toward target epithelial cells in the stomach mucosa, which is an essential requirement for subsequent cell invasions (Martins et al., 2009).

We also found that the sphingolipid metabolism pathway is enriched in the TcY-L1/2^−/−^ membrane subproteome from the upregulated proteins (**Supplementary Table S3**), therefore, indicating that this pathway was likely enriched in TcY-L1/2^−/−^ trypomastigotes in comparison to the TcY-WT trypomastigotes. We did not find upregulated proteins that would relate directly to the sphingolipid metabolism pathway (Booth and Smith, 2020). However, *T. cruzi* synthesizes the inositolphosphorylceramide as its primary phosphosphingolipid, which is also an attached lipid of GPI anchors (McConville and Ferguson, 1993; Lester and Dickson, 1993; Bertello et al., 1995; Uhrig et al., 1996; Figueiredo et al., 2005). Indeed, the proteins addressed to this pathway by the enrichment analysis, the UDP-Gal or UDP-GlcNAc-dependent glycosyltransferase (TcYC6_0105630) and five trans-sialidase members (TcYC6_0128720, TcYC6_0128910, TcYC6_0129770, TcYC6_0130520, and TcYC6_0131410), would relate better to the GPI-anchor pathway. N-Acetylglucosamine (GlcNAc) is transferred onto the acyl/alkyl-PI by glycosyltransferase complexes, and trans-sialidases are GPI-anchored proteins (Heise et al., 1996; Previato et al., 2004). Both the glycosylation process and the production of glycosylated proteins are known as important for mediating cellular recognition, host cell adhesion and invasion, and immune evasion by the parasite (Acosta-Serrano et al., 2001; Alves and Colli, 2007; Giorgi and de Lederkremer, 2011). Interestingly, TcY-L1/2^−/−^ trypomastigotes have the highest invasion rates in WT fibroblasts when compared to TcY-WT and TcY-L2^−/−^.

The present work provides evidence that the intracellular environment can modulate *T. cruzi*’s biological behavior through inducing changes on its surface molecules. The results described here led us to think that this intracellular modulation could be part of *T. cruzi*’s adaptability and ability to infect different cell types. In turn, the surface molecules modulation could contribute to *T. cruzi*’s tropism to host specific tissues and perhaps to immune system evasion as well. Evidence to this hypothesis needs still to be addressed.

## Data Availability Statement

The datasets presented in this study can be found in online repositories. The names of the repository/repositories and accession number(s) can be found below. The mass spectrometry proteomics data have been deposited to the ProteomeXchange Consortium *via* the PRIDE (WHO, 2021) partner repository with the dataset identifier PXD028897.

## Author Contributions

Conceptualization: LA and HA. Data curation: LA and HA. Formal analysis: AO. Funding acquisition: LA. Investigation: AO, LR, JG, and WB. Methodology: LA, AO, HA, MB, VG, FK, and LR. Project administration: LA. Resources: LA, HA, TB, GM, and FK. Supervision: LA, HA, VG, and FK. Validation: AO. Visualization: AO. Writing—original draft: AO. Writing—review and editing: LA, AO, HA, MB, VG, FK, and LR.

## Funding

Below are the funding resources for the development of this research: Conselho Nacional de Desenvolvimento Científico e Tecnológico (CNPq) (MCTIC/CNPq No 28/2018—Universal 429635/2018-4) Fundação de Amparo à Pesquisa do Estado de Minas Gerais (FAPEMIG—APQ-02974-17), Coordenação de Aperfeiçoamento de Pessoal de Nível Superior (CAPES / PROEX). None of the fundings received allow for publication fees, the resources being destined only to the acquisition of reagents for the development of the methodological assays.

## Conflict of Interest

The authors declare that the research was conducted in the absence of any commercial or financial relationships that could be construed as a potential conflict of interest.

## Publisher’s Note

All claims expressed in this article are solely those of the authors and do not necessarily represent those of their affiliated organizations, or those of the publisher, the editors and the reviewers. Any product that may be evaluated in this article, or claim that may be made by its manufacturer, is not guaranteed or endorsed by the publisher.
