# Supplementary material for: Biological and Molecular Effects of Trypanosoma cruzi Residence in a LAMP-Deficient Intracellular Environment
Source: Front Cell Infect Microbiol. 2022 Jan 6;11:788482. doi: 10.3389/fcimb.2021.788482 (PMC8770540; doi:10.3389/fcimb.2021.788482)
Supplement: Supplementary file 1 [file DataSheet_1.docx]

**SUPPLEMENTARY MATERIAL**

**SUPPLEMENTARY FIGURE 1**

**
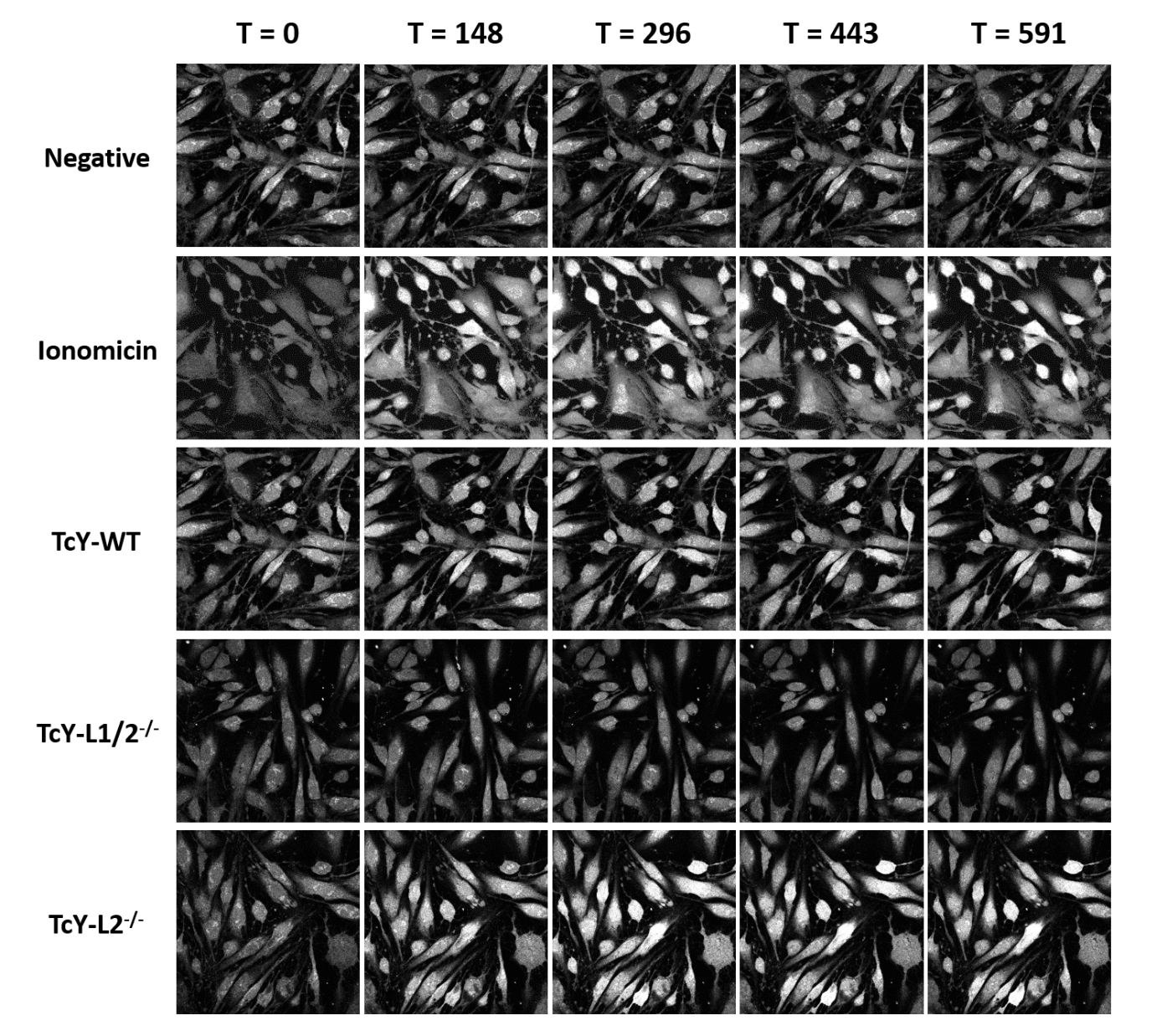
**

**Supplementary Figure 1:** **Calcium signaling assays in L6 myoblasts.** Time point representative confocal images from L6 myoblasts exposed to TcY-WT, TcY-L1/2^-/-^ and TcY-L2^-/-^ trypomastigotes, as described in the Materials and methods section. The L6 cells were also exposed to ionomicin as positive control and culture medium as negative control. T = time point in seconds.

**SUPPLEMENTARY FIGURE 2**

**
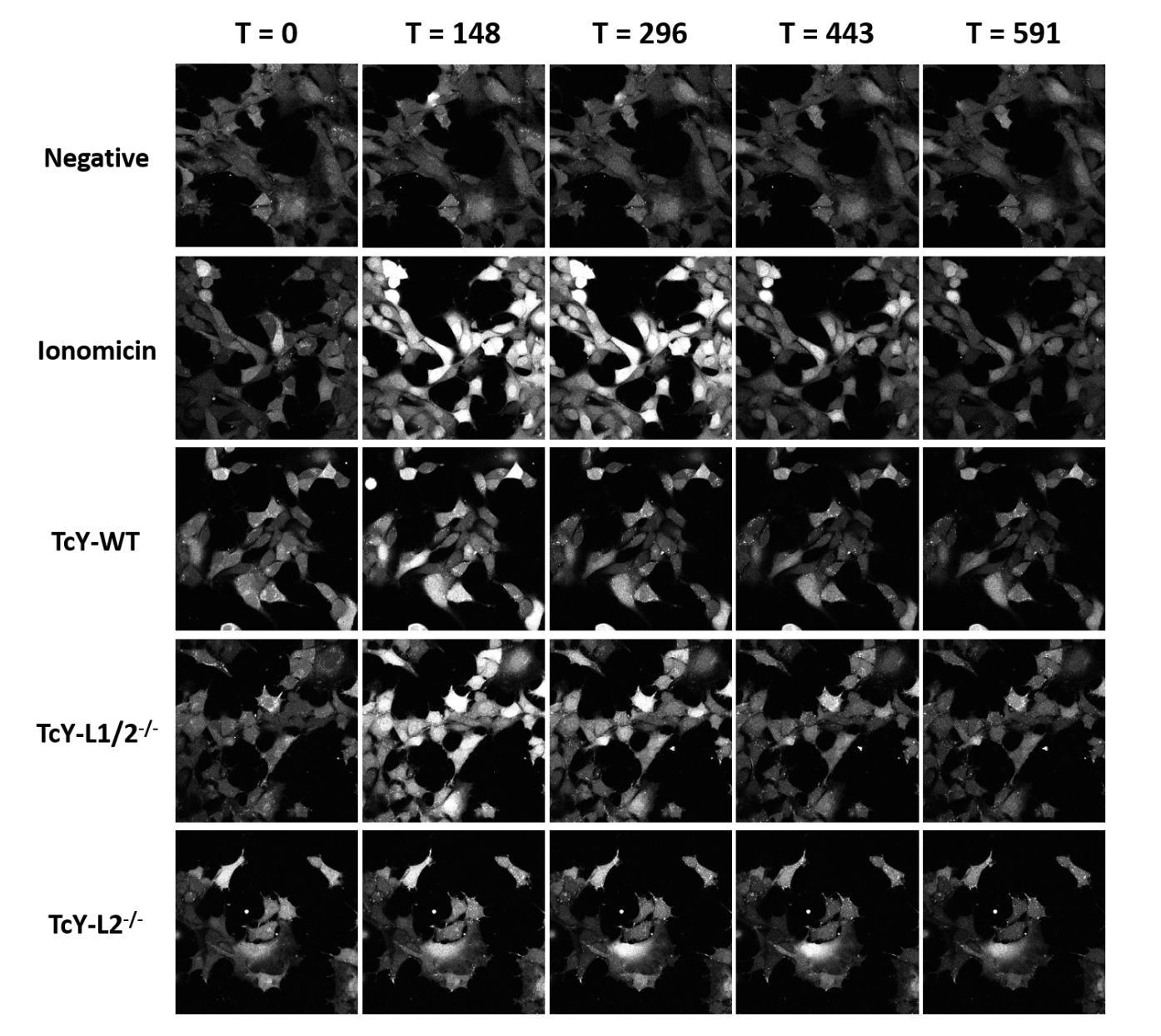
**

**Supplementary Figure 2:** **Calcium signaling assays WT fibroblasts.** Time point representative confocal images from WT fibroblasts exposed to TcY-WT, TcY-L1/2^-/-^ and TcY-L2^-/-^ trypomastigotes, as described in the Materials and methods section. The fibroblasts were also exposed to ionomicin as positive control and culture medium as negative control. T = time point in seconds.

**SUPPLEMENTARY FIGURE 3**


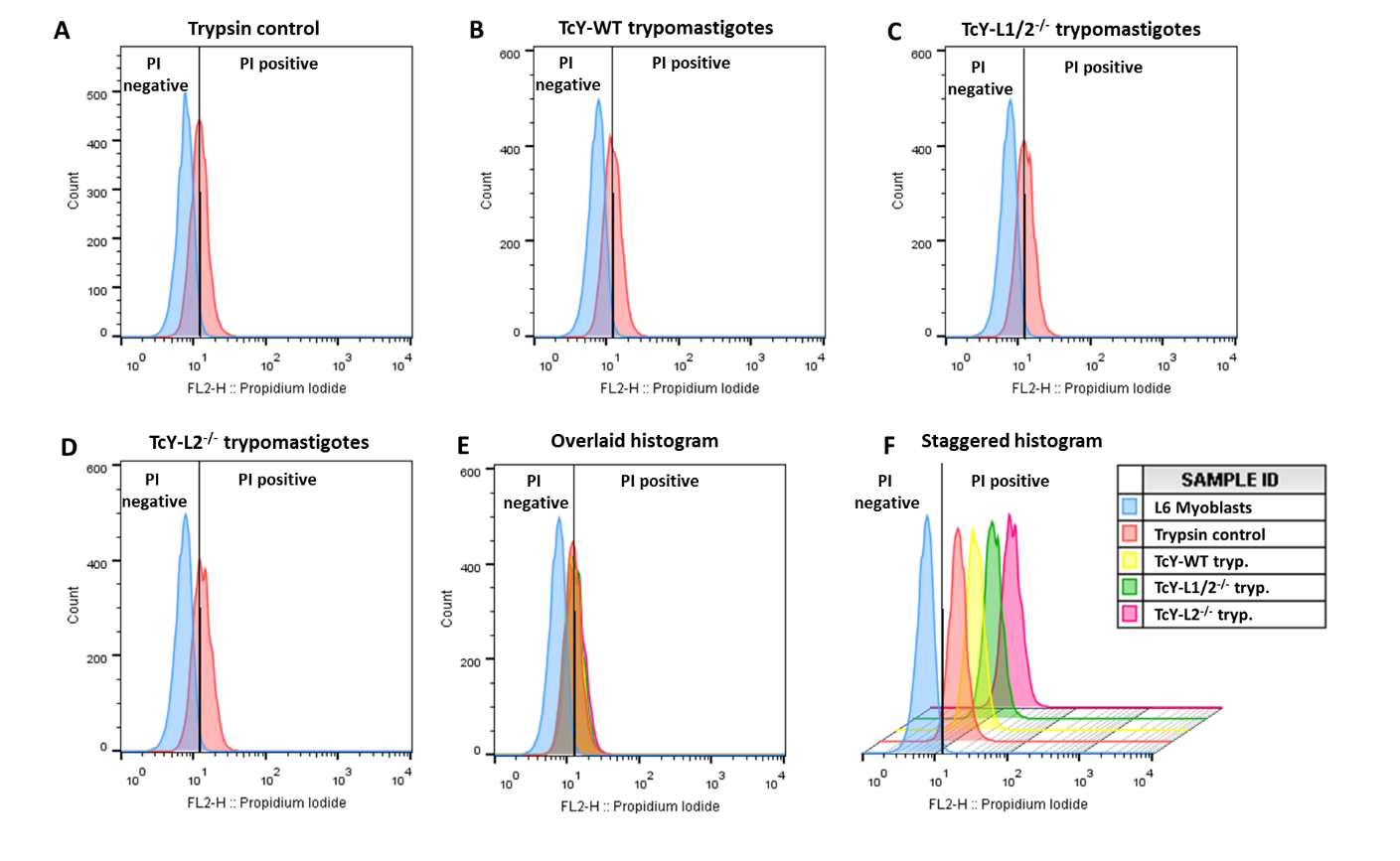


**Supplementary Figure 3: Membrane injury assays in L6 myoblasts.** The myoblasts were exposed to TcY-WT, TcY-L1/2^-/-^ and TcY-L2^-/-^ trypomastigotes in the presence of PI (propidium iodide) as described in the Materials and methods section. Representative histograms from the control - trypsinized cells only exposed to PI (A), TcY-WT (B), TcY-L1/2^-/-^ (C) and TcY-L2^-/-^ trypomastigotes (D). Overlaid (E) and staggered (F) histograms from all samples are also shown.

**SUPPLEMENTARY FIGURE 4**

**
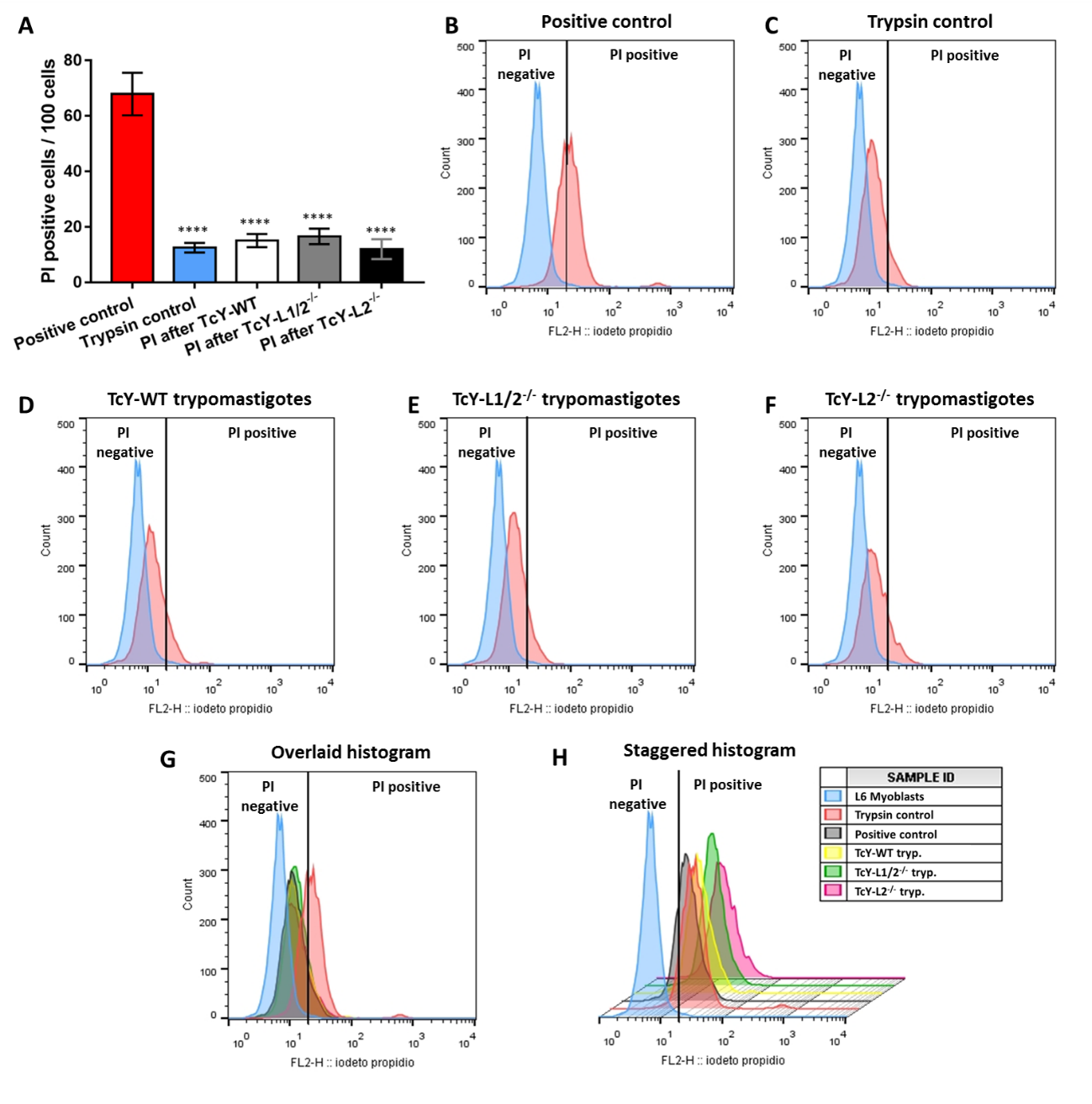
**

**Supplementary Figure 4: Membrane injury assays in L6 myoblasts.** The myoblasts were exposed to TcY-WT, TcY-L1/2^-/-^ and TcY-L2^-/-^ trypomastigotes for 40 min, washed three times and then exposed to PI (propidium iodide) for another 40 min, as described in the Materials and methods section. Membrane injury assay (A). Asterisks represent statistical difference compared to the positive control (**** p<0.0001, One-way ANOVA, FDR controlled by Benjamini and Hochberg). Representative histograms from the positive control – cells in suspension exposed to PI (B), trypsin control - trypsinized cells only exposed to PI (C), TcY-WT (D), TcY-L1/2^-/-^ (E) and TcY-L2^-/-^ trypomastigotes (F). Overlaid (G) and staggered (F) histograms from all samples are also shown.

**SUPPLEMENTARY FIGURE 5**

**
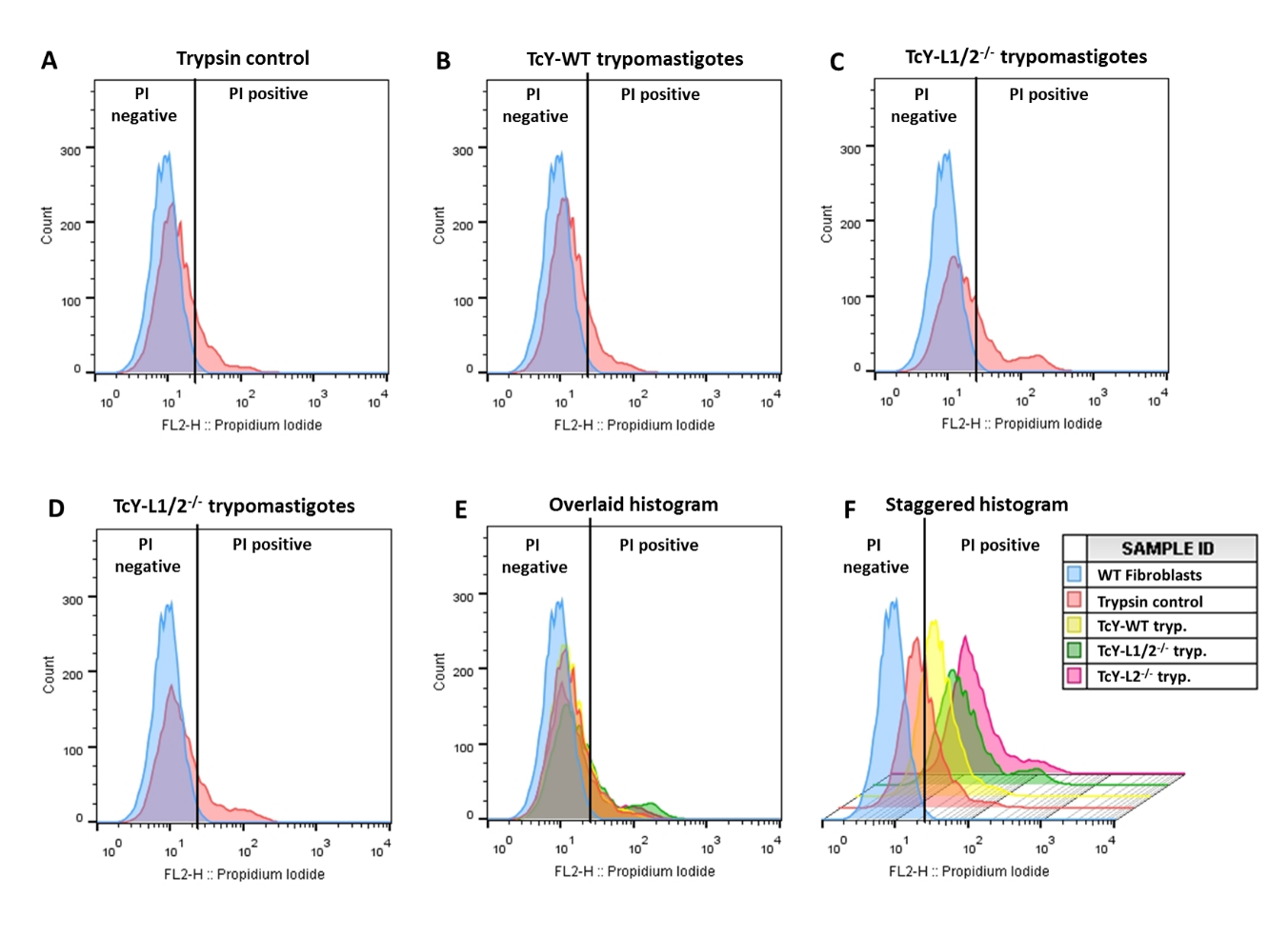
**

**Supplementary Figure 5: Membrane injury assays in WT fibroblasts.** The WT fibroblasts were exposed to TcY-WT, TcY-L1/2^-/-^ and TcY-L2^-/-^ trypomastigotes in the presence of PI (propidium iodide) as described in the Materials and methods section. Representative histograms from the control - trypsinized cells only exposed to PI (A), TcY-WT (B), TcY-L1/2^-/-^ (C) and TcY-L2^-/-^ trypomastigotes (D). Overlaid (E) and staggered (F) histograms from all samples are also shown.

**SUPPLEMENTARY FIGURE 6**


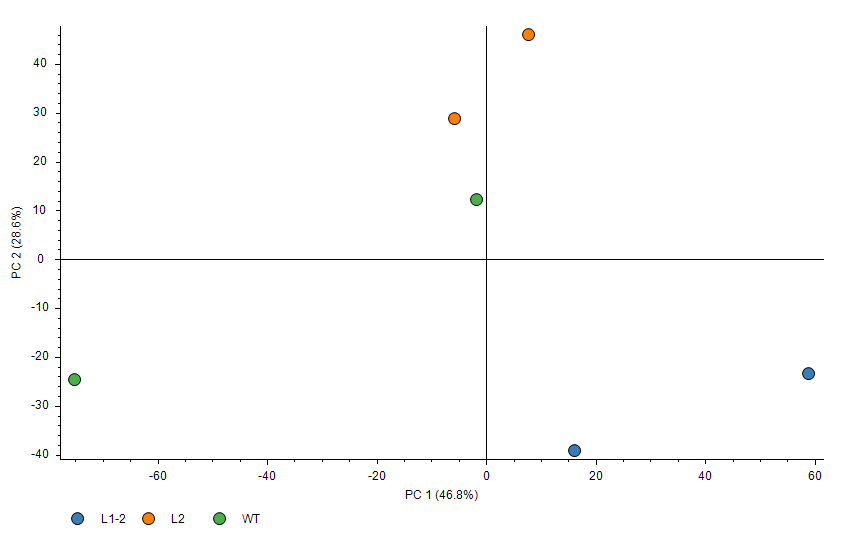


**Supplementary Figure 6:** Principal component analysis of the biological replicates from TcY-WT (green dots), TcY-L1/2^-/-^ (blue dots) and TcY-L2^-/-^ (orange dots) samples.

**SUPPLEMENTARY FIGURE 7**


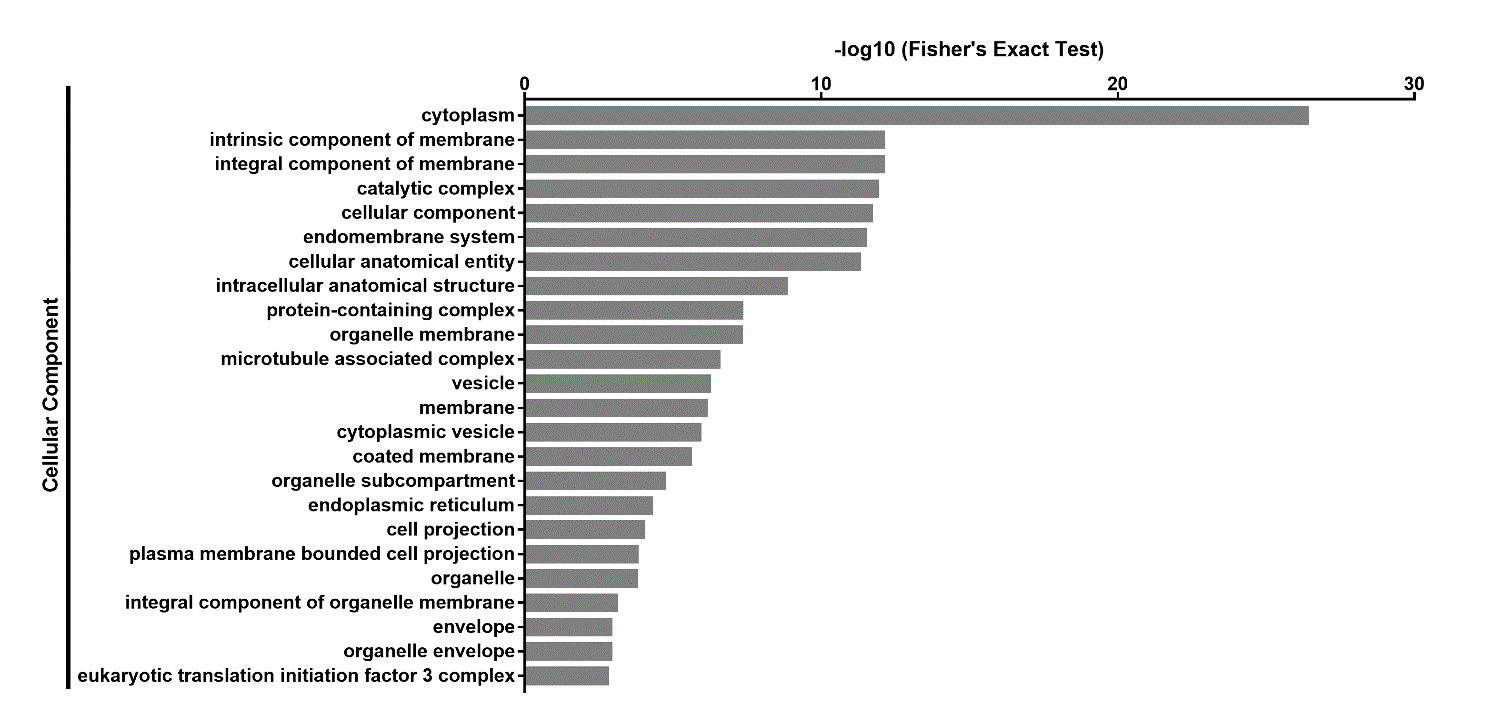


**Supplementary Figure 7: Gene ontology analysis of TcY-WT, TcY-L1/2^-/-^ and TcY-L2^-/-^ identified proteins confirmed that the samples were abundant in membrane terms.** The graph displays the cellular component level of the 3806 identified proteins submitted to Gene ontology enrichment analysis using the TriTrypDB gene ontology tool.

**SUPPLEMENTARY TABLE LEGENDS**

**Supplementary Table 1** – All identified proteins from TcY-WT, TcY-L1/2^-/-^ and TcY-L2^-/-^ proteomes. Table in Excel file format.

**Supplementary Table 2** – Regulated proteins from TcY-WT, TcY-L1/2^-/-^ and TcY-L2^-/-^ regulated subproteomes containing all the analysis performed. Table in Excel file format.

**Supplementary Table 3** – Gene Ontology Enrichment analysis for Biological Process terms and Metabolic Pathways analysis from the regulated proteins from TcY-WT, TcY-L1/2^-/-^ and TcY-L2^-/-^ subproteomes. Table in Excel file format.
